# Tailoring Substitutional Sites for Efficient Lanthanide Doping in Lead-Free Perovskite Nanocrystals with Enhanced Near-Infrared Photoluminescence

**DOI:** 10.1021/acsnano.5c00487

**Published:** 2025-04-11

**Authors:** Hanjie Lin, Sara Talebi, Walker MacSwain, Vanshika Vanshika, Arindam Chakraborty, Weiwei Zheng

**Affiliations:** Department of Chemistry, Syracuse University, Syracuse, New York 13244, United States

**Keywords:** lanthanide doping, substitutional sites, lead-free perovskites, nanocrystals, near-infrared photoluminescence

## Abstract

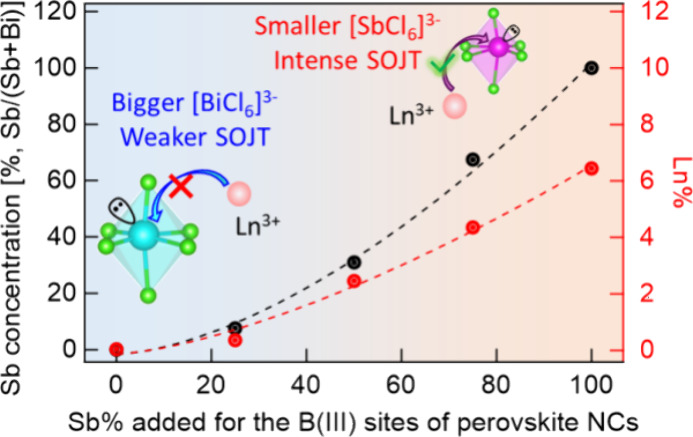

The incorporation of rare earth lanthanide ions (Ln^3+^) into lead-free halide perovskite nanocrystals (NCs) is an effective and promising strategy to expand their optical, magnetic, and electrochemical properties. Herein, we designed and synthesized various Ln^3+^ (including Yb^3+^, Er^3+^, and Nd^3+^), doped Sb^3+^- or Bi^3+^-based and Sb^3+^/Bi^3+^ alloyed lead-free perovskite NCs, including vacancy-induced perovskite (A_3_B(III)_2_X_9_), double perovskite (A_2_B(I)B (III)X_6_), and layered-double perovskite (A_4_B(II)B(III)_2_X_12_) NCs with different energy transfer pathways to study the Ln^3+^ dopant photoluminescence (PL). While a small size mismatch between dopant ions and host substitutional sites are critical for efficient doping of many first-row transitional metal ion doped metal chalcogenides, surprisingly, the Ln^3+^ ions, including the large Nd^3+^ ions (112 pm), prefer smaller isovalent Sb(III) octahedral (O_h_) sites (90 pm) instead of Bi(III) O_h_ sites (117 pm) in these lead-free perovskite NCs. Significantly, similar substitutional site-dependent Ln^3+^ doping efficiencies were obtained across all three different perovskite host lattices, despite differences in host-to-dopant energy transfer mechanisms, which can provide strong evidence of the preferred Sb^3+^ substitutional sites for lanthanide dopants in these lead-free perovskite lattices. The efficient Ln^3+^ doping in Sb^3+^-rich perovskite NCs leads to enhanced Ln^3+^ ion PL of the doped NCs. The preference of smaller Sb (III) over Bi(III) substitutional sites for Ln^3+^ dopants is attributed to the relatively high polarizabilities of lanthanide ions and the smaller cationic sites inside [SbX_6_]^3–^ compared with [BiX_6_]^3–^ octahedra. This study provides a fundamental understanding of Ln^3+^ doping behavior in lead-free perovskite NCs and opportunities for designing efficient Ln^3+^-doped functional materials by tuning the microenvironment of the host lattice for enhanced properties.

## Introduction

**Figure 1:**
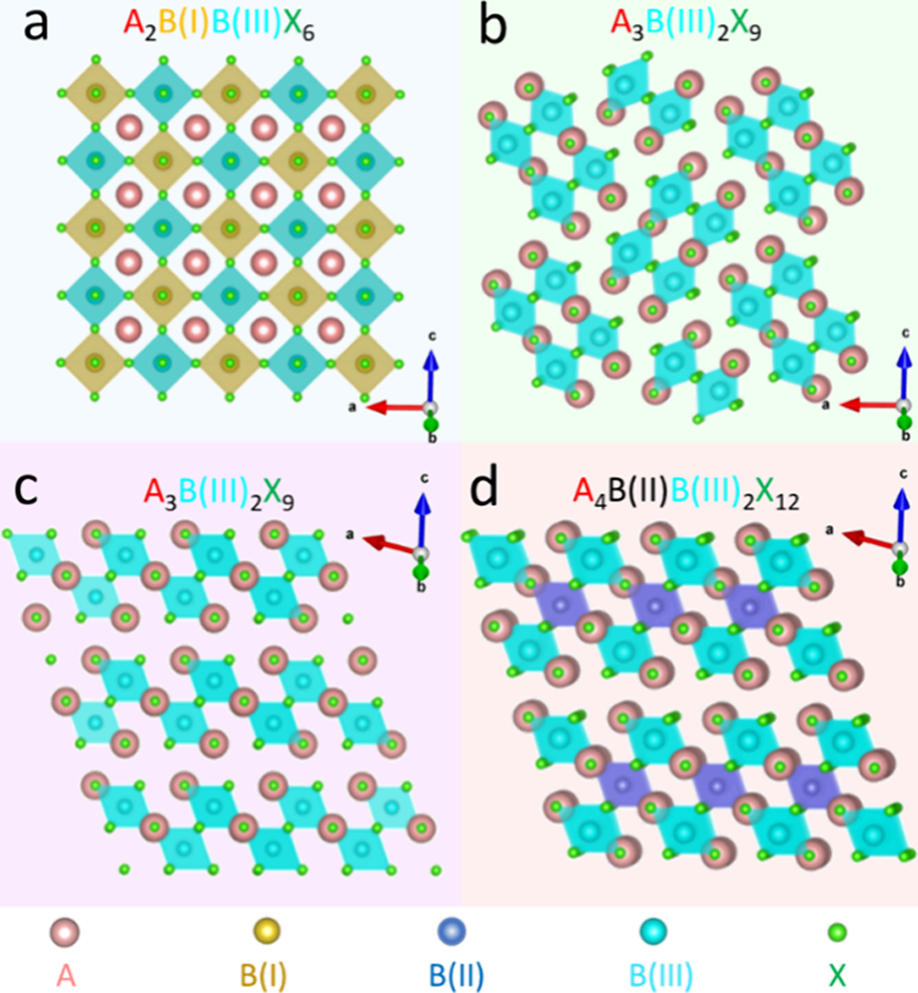
Crystal structures of (a) A_2_B(I)B(III)X_6_: cubic double perovskite, (b) A_3_B(III)_2_X_9_: orthorhombic perovskite, (c) A_3_B(III)_2_X_9_: trigonal 2D layered perovskite, and (d) A_4_B(II)B(III)_2_X_12_: trigonal 2D-layered double perovskite.

Lead halide (APbX_3,_ A = Cs^+^, Rb^+^; X = Cl^–^, Br^–^, I^–^) perovskite nanocrystals (NCs) have extraordinary properties including surface defect tolerance, high photoluminescence quantum yield (PL QY), and superior charge transport properties,^1−3^ which makes them great candidates for applications in light-emitting diodes (LEDs),^4−6^ photodetectors,^7^ solar cells,^8−11^ X-ray scintillators^12^ and photocatalysts,^13,14^*etc*. To address environmental and health concerns of the intrinsic toxicity of lead-based perovskites for broad practical applications, various lead-free perovskite derivatives with different elements and dimensionalities have been developed.^15,16^ A promising lead-free double perovskite was developed with the formula A_2_B^(I)^B^(III)^X_6_, which has the same 3-dimensional (3D) cubic structure as lead-based single perovskites, where two Pb^2+^ ions are replaced by a pair of one monovalent cation and one trivalent cation (Figure 1a).^17−20^ In addition, vacancy-induced perovskites with a formula of A_3_B_2_X_9_ have been developed (Figure 1b) such as Cs_3_Bi_2_X_9_, Cs_3_Sb_2_X_9_, and MA_3_Bi_2_X_9_ where the three Pb^2+^ ions are replaced by two B^3+^ cations and a vacancy.^21−23^ Additionally, if the A, B, and X sites are small enough, a layer of vacancies can be formed (Figure 1c).^24^ Furthermore, inserting a layer of [B^(II)^X_6_]^4–^ between the two layers of [B^(III)^X_6_]^3–^ in an A_3_B_2_X_9_ layered perovskite leads to layered double perovskites with a chemical formula of A_4_B^(II)^B^(III)^_2_X_12_ (B^(II)^: Cu^2+^, Cd^2+^, Mn^2+^, Sn^2+^, Zn^2+^; B^(III)^: Bi^3+^, Sb^3+^, In^3+^) (Figure 1d).^25−28^ These lead-free perovskites offer great flexibility in selecting the B-site cation elements and dimensionalities, along with decent stabilities and reduced toxicity. As a result, they are promising materials for potential applications in solar cells, LEDs, and biosensing. For example, Cs_4_CuSb_2_Cl_12_ has a low bandgap of 1.0 eV, which is very useful for applications of solar cells and photocatalysis;^25,26^ while Cs_4_MnBi_2_Cl_12_ exhibits a large bandgap with a strong orange emission from Mn ions and therefore has a large Stoke’s shift, which is promising for LEDs.^28^

Doping impurity ions is an effective way to manipulate the electronic, optical, and magnetic properties of semiconductor NCs.^29−43^ Due to the presence of 4f-electrons and the various 4f-4f intra-atomic electron transitions, lanthanide ion dopants usually have multiple sharp absorptions and PL peaks that can range from ultraviolet (UV) to near-infrared (NIR).^44^ In addition, the shielding effect of the 5s and 5p electrons keeps the 4f electrons from forming bonds with halide ions in lanthanide doped metal halide perovskites, resulting in stable 4f–4f transition energies and similar PL positions as those of the corresponding free ion.^32^ These properties make lanthanide-based and doped materials very promising for applications in LEDs,^45^ photodetectors,^46^ photocatalysts^47^ and anticounterfeiting.^48^

To date, there are several excellent works of doping lanthanide ions in lead-free perovskite NCs and bulk crystals.^49−54^ Lee et al. doped Yb^3+^ and Er^3+^ in Cs_2_AgInCl_6_ NCs with enhanced water stability compared to lead-based perovskites.^53^ However, very low PL QYs of the resulting lanthanide doped perovskite NCs (<1%) were reported. To improve the PL QYs of the lanthanide doped Cs_2_AgInCl_6_, researchers codoped Bi^3+^ or Sb^3+^ sites with lanthanide ions as optical sensitizers.^50,51,54^ For example, codoping Sb^3+^ with Yb^3+^ in Cs_2_AgInCl_6_ crystals can improve the NIR emission from Yb^3+^ by ∼ 140 times compared to singly doped Yb^3+^ in Cs_2_AgInCl_6_ crystals.^54^ Co-doping with Bi^3+^ can also alter the resulting optical properties, such as when Bi^3+^ is doped alongside Tb^3+^ ions in Cs_2_AgInCl_6_ NCs, resulting in a new excitation peak at ∼ 370 nm from the Tb^3+^ emission.^51^ In addition, Yb^3+^ and Er^3+^ were first doped in a layered double perovskite Cs_4_MnSb_2_Cl_12_ by Cai et al.,^38^ and the energy transfer pathways from host to Ln^3+^ dopants were studied in great detail. Nevertheless, there are only a few studies on lanthanide doped Sb^3+^/Bi^3+^-based perovskites,^49,55−57^ and a systematic study of preferred sites and microenvironment of lanthanide dopants in lead-free perovskite NCs has not been conducted.

Various dopants including many first-row transition metal ions, such as Mn^2+^ and Cu^+^, have been successfully doped in covalent metal chalcogenide NCs.^36,37,58−60^ In those doped II–VI NCs, the cationic radius mismatch between dopant ions and the host cationic site has great impact on the doping efficiency in the host lattice.^37,59^ For example, Nag et al. showed an increased Mn^2+^ doping efficiency within alloyed Zn_0.49_Cd_0.51_S NCs host lattice with a smaller size mismatch between Mn^2+^ and the alloyed host metal site, compared with that in either pure CdS or ZnS NCs.^37^ Considering the intermediate ionic radius of Mn^2+^ (80 pm) between Cd^2+^ (92 pm) and Zn^2+^ (74 pm), these results can be attributed to the reduced size mismatch between Mn^2+^ and alloyed host lattices. Our previous work found that directional Mn^2+^ dopant migration could occur from the initial substitutional sites to an alloyed CdZnS dopant “trap” layer with a smaller cationic size mismatch with dopant ions in Mn^2+^ doped core/shell and core/multishell quantum dots (QDs).^59,61^ However, Ln^3+^ dopant incorporation and diffusion behavior in a NC lattice has been less studied, in part due to relatively fewer studies on Ln^3+^ doped metal chalcogenide NCs as the fact that the large Ln^3+^ ions prefer substitutional sites with a large coordination number (CN) ≥ 6, such as O_h_ sites (CN = 6), instead of smaller tetrahedral (CN = 4) cationic sites in a host lattice.^30^ While Ln^3+^ dopants can be incorporated into many B-sites (O_h_ sites) in different types of perovskite NCs, a systematic study of doping efficiency and preferred doping sites of lanthanide ions in lead-free perovskite NCs is still unexplored.

In this study, we successfully synthesized a series of lanthanide doped Sb^3+^/Bi^3+^-based lead-free double perovskite (Cs_2_NaSb_*x*_Bi_1–*x*_Cl_6_), vacancy-induced perovskite (Cs_3_Sb_2*x*_Bi_2(1-x)_Cl_9_) and layered double perovskite (Cs_4_MnSb_2*x*_Bi_2(1-x)_Cl_12_) NCs by a hot-injection method. Because the ionic radii of all Ln^3+^ ions (from 101 to 117 pm) sit between Sb^3+^ (90 pm) and Bi^3+^ (117 pm),^62^ an alloyed Sb^3+^/Bi^3+^ alloyed perovskite could provide a smaller size mismatch with Ln^3+^ compared with either pure Sb^3+^- or Bi^3+^-based perovskites. Surprisingly, despite using the same amount of lanthanide precursors in all syntheses, the Bi^3+^-based perovskites showed negligible lanthanide emissions and lanthanide doping, while the pure Sb^3+^-based perovskite hosts show the strongest Ln^3+^ PL and highest Ln^3+^ doping efficiency. In addition, as there is an increase of Sb^3+^ concentration in the Sb^3+^/Bi^3+^ alloyed perovskite NCs, the PL intensity of lanthanide ions increases monotonically. Elemental analysis by ICP-OES also shows that more Ln^3+^ ions were incorporated into the lattice of the perovskite NCs with an increase in Sb^3+^ content. Significantly, the trends of the Ln^3+^ doping concentration for Sb^3+^/Bi^3+^ alloyed NCs follow the Sb^3+^ content of the Sb^3+^/Bi^3+^ alloyed NCs, which strongly indicates that Sb^3+^ provides preferred substitutional doping sites for Ln^3+^ dopants when compared to Bi^3+^. While enhanced doping efficiency of first-row transition metal ion dopants in metal chalcogenide host NCs with a small cationic size mismatch between the host and dopant has been reported, we attribute our opposite trend for lanthanide dopants in the three perovskite NCs (Sb^3+^ with a smaller ionic radius allowing for more Ln^3+^ dopant incorporation compared to Bi^3+^) to the ionic nature of the perovskite and the higher polarizability of Ln^3+^ compared to many first-row transition metal dopant ions.^63^ In addition, the more significant second-order Jahn–Teller distortion in [SbX_6_]^3–^ octahedra compared to [BiX_6_]^3–^ octahedra might also facilitate the incorporation of Ln^3+^ ions. This work indicates that for large Ln^3+^ dopants, the size mismatch between host and dopant is not the most critical parameter for doping efficiency which is opposite to smaller first-row transition metal dopants. The highly polarizable large lanthanide dopant ions prefer relatively small O_h_ substitutional sites found, for example, in an ionic perovskite host lattice. The findings from this work provide deeper understanding of dopant behaviors in nanoscale crystals.

## Results and Discussion

### Yb^3+^-Doped Cs_2_NaSb_*x*_Bi_1–*x*_Cl_6_ Double Perovskite NCs

Colloidal syntheses of Ln^3+^ doped lead-free perovskite NCs were conducted following a reported hot-injection method (see synthetic details in the Experimental section).^64−67^ Briefly, metal acetates for the host perovskites and lanthanide acetate(s) as the dopant resource (20% by mole of the (Sb^3+^ + Bi^3+^) precursors) were added into a mixture of oleic acid (OA), oleylamine (OAm), and 1-octadecene (ODE). For Yb^3+^ doped Cs_2_NaSb_*x*_Bi_1–*x*_Cl_6_ double perovskite NCs, a 20% feeding ratio of Yb^3+^ precursor was chosen as it produces the most intense Yb^3+^ PL of the doped NCs (5–30% mol ratio of Yb^3+^ precursor is shown in Figure S1) and a 20% mol ratio was used for other Ln^3+^ doped lead-free perovskite NCs in this study. After complete dissolution of all metal acetates, chlorotrimethylsilane (TMS-Cl), which served as an anion precursor, was rapidly injected in the mixture at high temperatures (170 – 200 °C) to initiate the nucleation of the NCs. Then, the reaction was quenched by a water bath after 5 s – 5 min.

**Figure 2:**
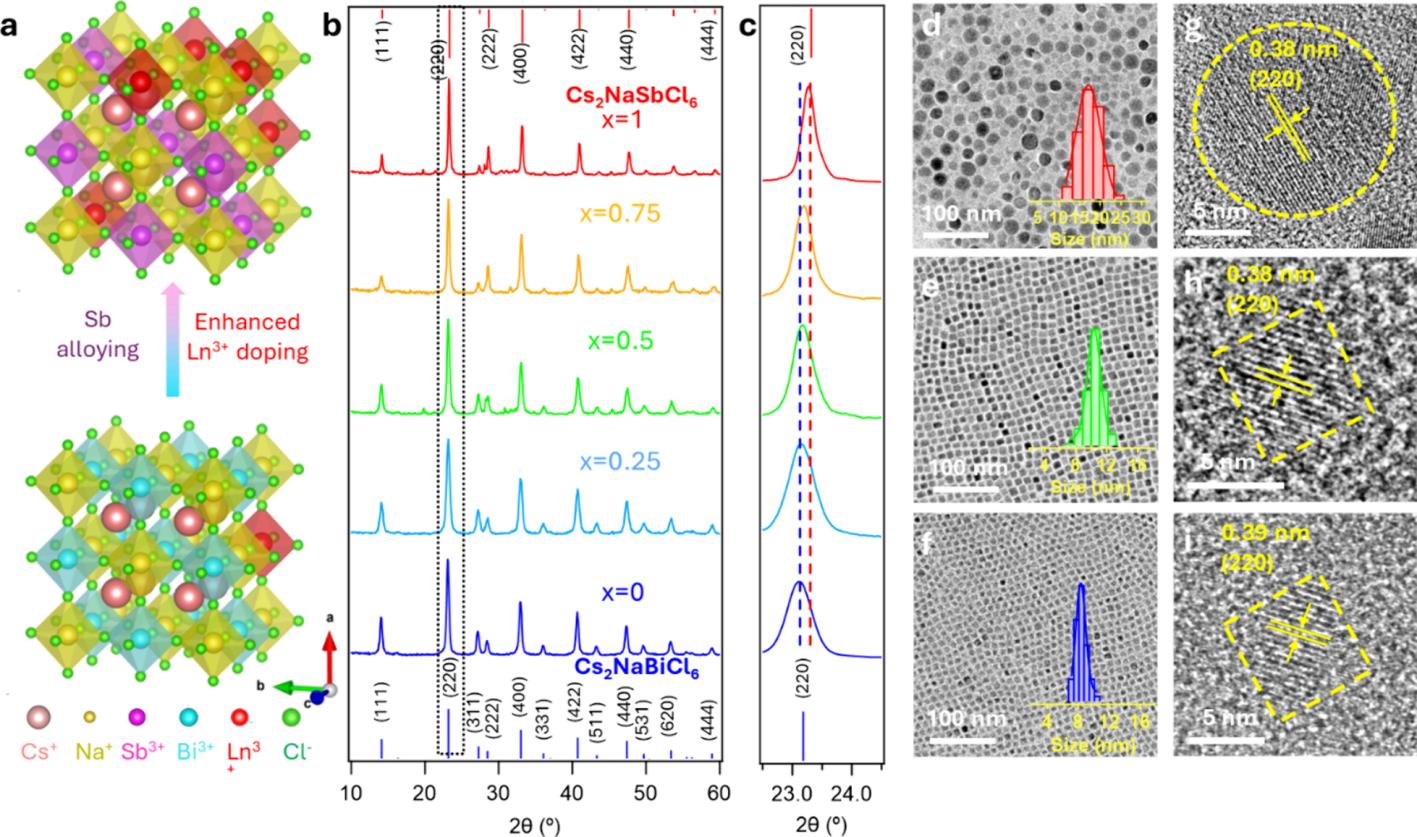
(a) Crystal structure of Yb^3+^-doped Cs_2_NaSbCl_6_ and Cs_2_NaBiCl_6_ NCs. (b) XRD pattern of Yb^3+^-doped Cs_2_NaSb_*x*_Bi_1–*x*_Cl_6_ NCs. (c) Zoomed-in XRD patterns showing peak shifting of the (220) diffraction peak by alloying Sb^3+^ into Cs_2_NaBiCl_6_ NCs. TEM images of (d) Yb^3+^-doped Cs_2_NaSbCl_6_ NCs, (e) Yb^3+^-doped Cs_2_NaSb_0.5_Bi_0.5_Cl_6_ NCs, and (f) Yb^3+^-doped Cs_2_NaBiCl_6_ NCs, with the insets being respective size distribution histogram. High-resolution TEM images of (g) Yb^3+^-doped Cs_2_NaSbCl_6_ NCs, (h) Yb^3+^-doped Cs_2_NaSb_0.5_Bi_0.5_Cl_6_ NCs and (i) Yb^3+^-doped Cs_2_NaBiCl_6_ NCs.

Yb^3+^ doped Cs_2_NaSbCl_6_ and Cs_2_NaBiCl_6_ NCs exhibited a cubic phase with the space group of Fm3̅m, in which the [NaCl_6_]^5–^ octahedra share their corners with [BiCl_6_]^3–^ or [SbCl_6_]^3–^ octahedra and the Cs^+^ sit in the void of 8 octahedra (Figure 2a). Figure 2b-c shows the X-ray diffraction (XRD) patterns of Yb^3+^ doped lead-free Cs_2_NaSb_*x*_Bi_1–*x*_Cl_6_ NCs. For the Yb^3+^ doped alloyed Cs_2_NaSb_*x*_Bi_1–*x*_Cl_6_ NCs, no new phase is observed compared to the undoped Cs_2_NaSb_*x*_Bi_1–*x*_Cl_6_ NCs (Figure 2b), indicating the unaltered host structure of the Yb^3+^ doped Cs_2_NaSb_*x*_Bi_1–*x*_Cl_6_ NCs. With increasing the Sb^3+^ content in Cs_2_NaSb_*x*_Bi_1–*x*_Cl_6_ NCs, the (220) diffraction peak shifts from 23.10° to 23.28° (Figure 2c). This result indicates a trend of lattice shrinkage upon replacing Bi^3+^ (117 pm) ions with smaller Sb^3+^ (90 pm) ions. XRD patterns of control experiments of undoped Cs_2_NaSb_*x*_Bi_1–*x*_Cl_6_ NCs (Figure S2a) also show a continuous shift of the (220) diffraction peak from 23.10° to 23.33° upon increasing the concentration of Sb^3+^ ions (Figure S2b), which further confirms the Sb^3+^ alloying in the NCs.

Figure 2d-f show TEM images of the as-synthesized Yb^3+^ doped Cs_2_NaSb_*x*_Bi_1–*x*_Cl_6_ perovskite NCs. Yb^3+^ doped Cs_2_NaSbCl_6_, Cs_2_NaSb_0.5_Bi_0.5_Cl_6_, and Cs_2_NaBiCl_6_ NCs showed monodispersed nanoparticles with a size of 17.5 ± 4.0 nm, 10.2 ± 1.1 nm and 8.3 ± 0.9 nm, respectively (Figure 2d-f). The morphology change, from nanocubes to nanospheres, for Cs_2_NaBiCl_6_ to Cs_2_NaSbCl_6_ might be due to different nucleation and growth kinetics.^68^ The high-resolution TEM image of Yb^3+^ doped Cs_2_NaBiCl_6_ NCs shows a clear lattice fringe of 0.39 nm, which can be assigned to the (220) planes of the Cs_2_NaBiCl_6_ (Figure 2i). For the Yb^3+^ doped Cs_2_NaSbCl_6_ NCs, a lattice shrinkage from 0.39 to 0.38 nm was observed due to the replacement of Bi^3+^ by smaller Sb^3+^, which is consistent with the shift of the XRD peaks of the alloyed NCs. (Figure 2g). The corresponding FFT images of these HR-TEM images are shown in Figure S3 a-f.

**Figure 3:**
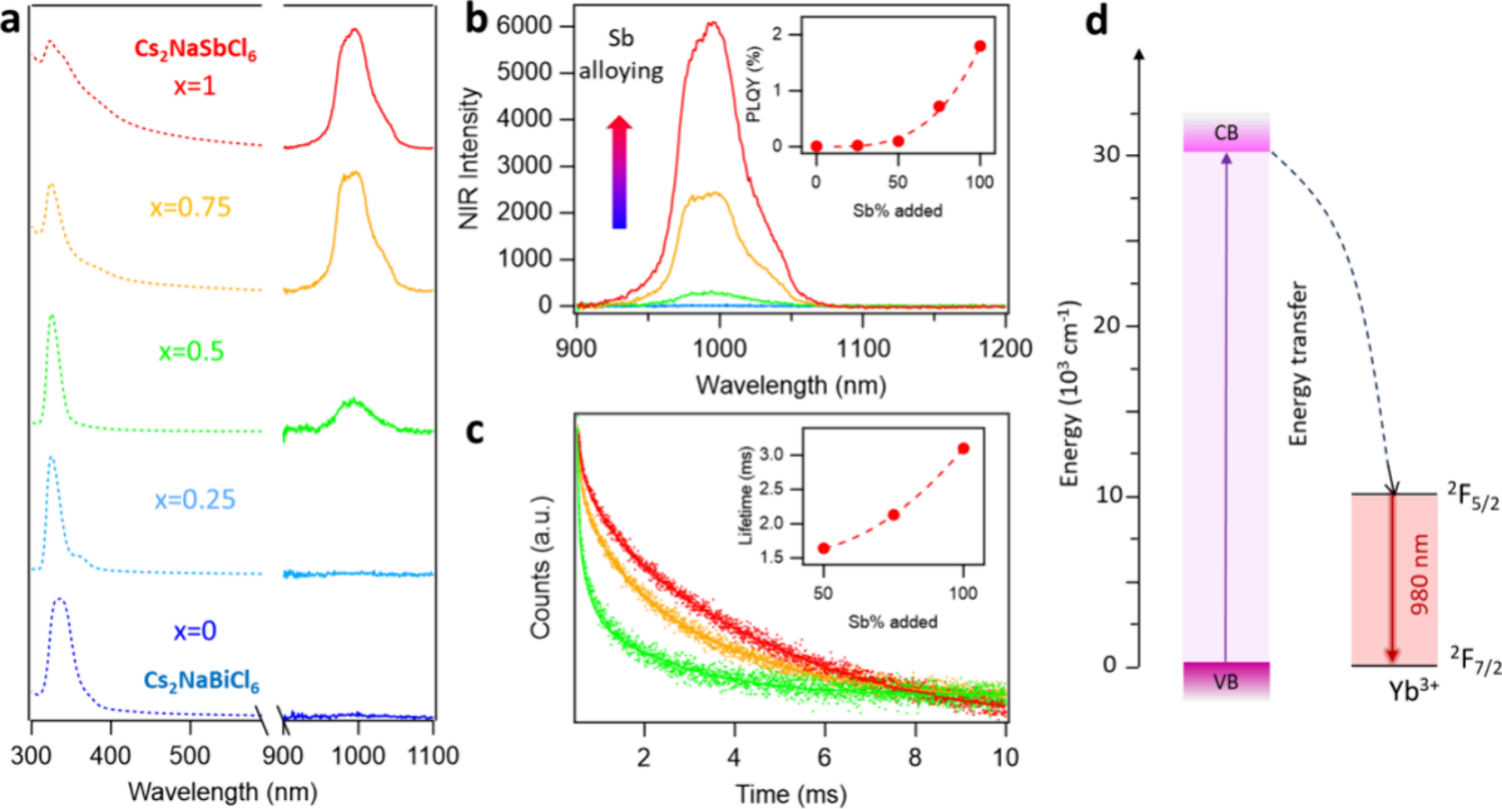
(a) Normalized absorption and NIR PL emissions of the Cs_2_NaBi_1–*x*_Sb_*x*_Cl_6_ from (*x* = 0 to 1). (b) NIR PL emissions spectra with the actual intensity and (inset) NIR PL QYs vs Sb% (x = 0 to 1) for the Yb^3+^-doped Cs_2_NaSb_*x*_Bi_1–*x*_Cl_6_ NCs. (c) Time-resolved photoluminescence (TR-PL) spectra and average PL lifetime (inset) of Yb^3+^-doped Cs_2_NaSb_*x*_Bi_1–*x*_Cl_6_ (*x* ≥ 0.5). (d) Band alignment and electronic state energy levels for the perovskites host and dopant ions.

The absorption and NIR PL spectra of Yb^3+^ doped Cs_2_NaSb_*x*_Bi_1–*x*_Cl_6_ NCs are shown in Figure 3a. An absorption peak at ∼ 335 nm was observed for Yb^3+^ doped Cs_2_NaBiCl_6_ NCs, which can be assigned to the 6s^2^→6s^1^6p^1^ transitions of Bi^3+^ in the [BiX_6_]^3–^ octahedron.^65^ The Yb^3+^ doped Cs_2_NaSb_*x*_Bi_1–*x*_Cl_6_ NCs exhibit a similar absorption peak due to the same valence shell electronic configuration of Sb^3+^ and Bi^3+^ (*ns*^2^).^69^ Therefore, we believe that the optical bandgap remains largely unchanged after incorporating Sb^3+^ into the Cs_2_NaBiCl_6_ NCs. An increased absorption tail on the right side of the absorption peak was observed when more Sb^3+^ is incorporated into Yb^3+^ doped Cs_2_NaSb_*x*_Bi_1–*x*_Cl_6_ NCs, probably due to the phonon-assisted absorption process in Sb^3+^-based perovskites.^70,71^ Yb^3+^ doping has been proven to be a way to tune the optical properties of NCs,^32,53^ as the f-f transition (^2^F_5/2_ to ^2^F_7/2_) in Yb^3+^ ions provides a new PL located at ∼ 990 nm for the doped NCs (Figure 3d). Interestingly, the Yb^3+^ doped Cs_2_NaBiCl_6_ NCs have negligible Yb^3+^ PL in the NIR range, however, as more Sb^3+^ is incorporated into the host material, the Yb^3+^ PL intensity exhibits an exponential growth (Figure 3b), reaching a 1.8% NIR PL QY for Yb^3+^ doped Cs_2_NaSbCl_6_ (Table S1). In addition, the lifetime of the Yb^3+^ emission at ∼ 990 nm shows similar exponential growth trend as the strength of the emissions (Figure 3c), increasing from 1.53 ms for Yb^3+^ doped Cs_2_NaSb_0.5_Bi_0.5_Cl_6_ NCs to 3.08 ms for Yb^3+^ doped Cs_2_NaSbCl_6_ NCs. The optical data indicates that Yb^3+^ ions might prefer to occupy a smaller [SbCl_6_]^3–^ octahedron site than a larger [BiCl_6_]^3–^ octahedron site in the lead-free Cs_2_NaSb_*x*_Bi_1–*x*_Cl_6_ double perovskite NCs.

**Figure 4:**
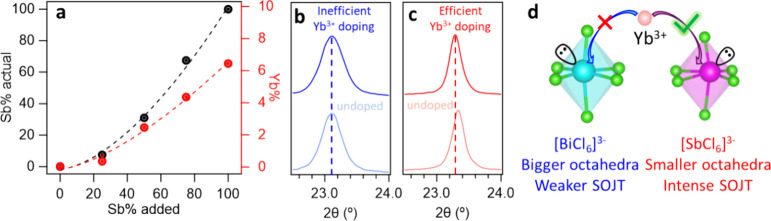
(a) The actual concentration of Sb^3+^ and Yb^3+^ in Yb^3+^-doped Cs_2_NaSb_*x*_Bi_1–*x*_Cl_6_ nanocrystals by ICP-OES. Zoom-in XRD patterns of Yb^3+^-doped and undoped (b) Cs_2_NaBiCl_6_ NCs and (c) Cs_2_NaSbCl_6_ NCs. (d) Schematic illustration of higher lanthanide doping efficiency in Cs_2_NaSbCl_6_ compared to Cs_2_NaBiCl_6_ based on Cs_2_NaSbCl_6_ having smaller octahedra and a more intense second-order Jahn–Teller distortion (SOJT).

To further study the doping efficiency of lanthanide ions in the lead-free perovskite NCs, we conducted ICP-OES measurements (Figure 4). The actual Sb^3+^ content ([Sb^3+^]/([Sb^3+^]+[Bi^3+^])) in B-site doped alloyed perovskite NCs shows an exponential growth rather than linear growth as a function of Sb precursor, Sb(OAc)_3_, added in the reactions. Importantly, the Yb^3+^ doping concentration ([Yb^3+^]/([Yb^3+^]+[Sb^3+^]+[Bi^3+^])) in Cs_2_NaSb_*x*_Bi_1–*x*_Cl_6_ NCs also exhibits an exponential growth as more Sb^3+^ is added (Figure 4a). The concentration of Yb^3+^ in Cs_2_NaBiCl_6_ NCs is nearly zero. This low doping efficiency of lanthanide ions in Bi-based perovskite NCs was also reported in Yb^3+^ doped Cs_2_AgBiBr_6_ NCs.^72^ When Bi^3+^ is completely replaced by Sb^3+^, the concentration of Yb^3+^ improves to 6.4% in Yb^3^ doped Cs_2_NaSbCl_6_ NCs. A similar trend of the concentration of Sb^3+^ and Yb^3+^ inside the perovskite NCs suggests that adding Sb^3+^ into the NC lattice can enhance the Yb^3+^ doping, indicating the Sb^3+^ in Sb-rich perovskites can provide better substitutional sites than Bi^3+^ for Yb^3+^ dopants.

Yb^3+^ doping into the Sb^3+^-site of the Cs_2_NaSb_*x*_Bi_1–*x*_Cl_6_ NCs can be further proven by XRD measurements. Comparing the XRD pattern of Yb^3+^ doped and undoped Cs_2_NaSbCl_6_ NCs, the (220) diffraction peak shifts to lower angle upon Yb^3+^ doping (Figure 4c) due to the larger ionic radii of Yb^3+^ (101 pm) than Sb^3+^ (90 pm).^38^ However, the (220) diffraction peak does not show an obvious shift after adding Yb^3+^ dopant precursors into the synthesis of Cs_2_NaBiCl_6_ NCs (Figure 4b), indicating inefficient/unsuccessful doping in Cs_2_NaBiCl_6_ host NCs. This result contradicts doping Mn into metal chalcogenide NCs, in which enhanced doping efficiency was achieved when the size mismatch between host and dopant is minimized. In Yb^3+^ doped Cs_2_NaSb_*x*_Bi_1–*x*_Cl_6_ NCs, the size mismatch between Yb^3+^ dopants (101 pm) and either larger Bi^3+^ (117 pm) or smaller Sb^3+^ (90 pm) substitutional sites is 13.6% and 12.2% for Yb^3+^ doped Cs_2_NaBiCl_6_ and Cs_2_NaSbCl_6_, respectively. The small difference of those two lattice mismatches is unlikely to be the major reason for the big difference in doping efficiency in those perovskite NCs. Based on the Vegard’s Law,^73^ the size of the B(III) site of x = 0.25 (0.075 for the NCs from ICP data), 0.5 (0.31 from ICP data), and 0.75 (0.67 from ICP data) Cs_2_NaSb_*x*_Bi_1–*x*_Cl_6_ NCs should be 115.0, 108.6, and 98.9 pm, with cationic size mismatch of 12.1%, 7.0% and 2.1%, respectively (Figure S4). Therefore, the alloyed Cs_2_NaSb_*x*_Bi_1–*x*_Cl_6_ host would provide a much smaller size mismatch compared to either pure Cs_2_NaSbCl_6_ (12.2%) and Cs_2_NaBiCl_6_ NCs (13.6%). However, the Yb^3+^ doping efficiency is lower in alloyed Cs_2_NaSb_*x*_Bi_1–*x*_Cl_6_ compared to that in pure Cs_2_NaSbCl_6_ and the Yb^3+^ doping efficiency increases with higher Sb^3+^ concentration in the alloyed NCs. Therefore, we attributed this result to the relatively high polarizability of large Ln^3+^ (from 2.630 to 3.920 Å^3^).^63^ Compared to smaller first-row transition metal ions, such as Mn^2+^ (polarizability 2.074 Å^3^), Ln^3+^ are larger and softer, which can allow the Ln^3+^ ions to insert themselves into smaller host O_h_ sites. In addition, the cation would prefer to occupy a smaller site in an anionic compound, by slightly expanding its structure. However, a larger doping site, in which the cation may “rattle” inside its anion polyhedron, is considered to be unstable.^74^ The lattice expansion due to Yb^3+^ doping into smaller Sb^3+^ sites is observed in XRD measurements (Figure 4c). In our previous work, Mn^2+^ is very stable when doped inside a smaller Zn^2+^ site in the ZnS lattice, which can prevent dopant diffusion and migration under elevated temperatures.^58^

Furthermore, both Sb^3+^ ([Kr]4d^10^5s^2^) and Bi^3+^ ([Xe] 4f^14^5d^10^6s^2^) possess an electron configuration of *n*s^2^ in their valence shell; it is reported that the accommodation of a lone pair of s^2^ electrons in an s-type antibonding orbital originates from the interaction between the cation s orbital and anion p orbital in [SbCl_6_]^3–^ and [BiCl_6_]^3–^ octahedra leading to a nonsymmetrical coordination environment.^75,76^ The resulting second-order Jahn–Teller (SOJT) O_h_ distortion is more intense in Cs_2_NaSbCl_6_ than that in Cs_2_NaBiCl_6_, probably due to the more diffuse character of the 6s^2^ electron density (more delocalized 6s^2^ electrons) in [BiCl_6_]^3–^. Cao et al. reported that the distortion of the inorganic octahedra in (TMEDA)_3_(Sb_0.5_Bi_0.5_)_2_Cl_12_H_2_O is significantly larger than that in unalloyed (TMEDA)_3_Bi_2_Cl_12_H_2_O.^77^ The more intense distortion in [SbCl_6_]^3–^ might facilitate the incorporation of lanthanide ions (Figure 4d).

To exclude the different doping efficiency caused by the different activity of Sb^3+^ and Bi^3+^ precursors in the nucleation process,^35^ doping experiments by cation exchange were conducted (Figure S5). An excess amount of YbCl_3_ with Yb/(Sb + Bi) = 100% was mixed with undoped Cs_2_NaSb_*x*_Bi_1–*x*_Cl_6_ NCs (x = 0, 0.25, 0.5, 0.75 and 1) in toluene, followed by stirring the mixture for 15 h (see detailed information in Supporting Information).^78^ We used 100% Yb_3+_ for the cation exchange, rather than the 20% used in the direct synthesis method for doped NCs, because a 20% Yb^3+^ doping concentration led to insignificant Yb^3+^ incorporation after 15 h of cation exchange at room temperature.

For the cation exchange reaction doping with 100% Yb^3+^, the NIR intensities increase monotonically in Cs_2_NaSb_*x*_Bi_1–*x*_Cl_6_ as x increases from 0 to 1 with alloying from Sb^3+^ (Figure S5a, b); the increase is similar to the results observed in Yb^3+^ doped Cs_2_NaSb_*x*_Bi_1–*x*_Cl_6_ NCs synthesized by the direct hot-injection method. The ICP-OES results also show a similar increasing trend of Sb^3+^ and Yb^3+^ content between cation exchange doping and direct synthesis doping (Figure S5c), indicating the different Yb^3+^ doping efficiency in the Cs_2_NaSb_*x*_Bi_1–*x*_Cl_6_ NCs is mainly due to the different microenvironments of the substitutional sites (Sb^3+^vs Bi^3+^) instead of kinetically controlled during synthesis.

It should be noted that the activity of halide precursors can also influence the Ln^3+^ doping in the NCs. In a control experiment, we used benzoyl chloride as an alternative halide source to synthesize Yb^3+^ doped Cs_2_NaSb_*x*_Bi_1–*x*_Cl_6_ NCs, following the same experimental procedure as when TMS-Cl was used as the Cl precursor. The Yb^3+^ doped Cs_2_NaSbCl_6_ NCs synthesized with benzoyl chloride exhibited a higher [Yb^3+^] (10.0%) compared to those synthesized with TMS-Cl, likely due to the higher reactivity of benzoyl chloride compared to TMS-Cl.^80^ Upon the increase of Sb^3+^, both the PL from Yb^3+^ (Figure S6a, b) and the Yb^3+^ doping concentration measured by ICP-OES (Figure S6c) increased in the as-synthesized NCs. Therefore, the trend of Sb/Bi-dependent Yb^3+^ PL at ∼ 990 nm and doping concentration is similar to that observed in the NCs synthesized using TMS-Cl. These results indicate that while the anion precursors could influence in doping efficiency, Yb^3+^ still prefer Sb^3+^ sites, regardless of the choice of anion precursor.

The size of the doped Cs_2_NaSbCl_6_ NCs (17.5 ± 4.0 nm) is larger than that of Cs_2_NaBiCl_6_ NCs (8.3 ± 0.9 nm), and it is reported that more Yb^3+^ can be incorporated into larger CsPbCl_3_ NCs.^79^ To determine if there is an effect of the host NC size on the doping efficiency in lead-free perovskite NCs, a control experiment of Yb^3+^ doped Cs_2_NaBiCl_6_ NCs with similar size and shape to the Yb^3+^ doped Cs_2_NaSbCl_6_ NCs was conducted (Figure S7). The synthesis is the same as the synthesis of small Yb^3+^ doped Cs_2_NaBiCl_6_ NCs, but the temperature is elevated to 200 °C instead of 170 °C. The shape of the Yb^3+^ doped Cs_2_NaBiCl_6_ NCs is cuboctahedral with a size of 18.4 ± 3.0 nm (similar to the Yb^3+^ doped Cs_2_NaSbCl_6_ NCs, 17.5 ± 4.0 nm) (Figure 2d and S7a), consistent with the previous report,^65^ likely due to ripening under high temperatures. Interestingly, the NIR PL of Yb^3+^ is still negligible even in larger Yb^3+^ doped Cs_2_NaBiCl_6_ NCs (Figure S7c). In addition, the elemental analysis result from ICP-OES indicates that the Yb^3+^% was increased from 0.04% for smaller Yb^3+^ doped Cs_2_NaBiCl_6_ NCs (8.3 ± 0.9 nm) to 0.22% for Yb^3+^ doped larger Cs_2_NaBiCl_6_ NCs (18.4 ± 3.0 nm). Even with a similar size and shape as well as a higher energy to insert the dopant into the host lattice, the concentration of Yb^3+^ in similar sized Yb^3+^ doped Cs_2_NaBiCl_6_ NCs (0.22%, 18.4 ± 3.0 nm) is still much lower than that of Yb^3+^ doped Cs_2_NaSbCl_6_ NCs (6.4%, 17.5 ± 4.0 nm) indicating that the size of the host NCs is not main reason for the difference in doping efficiency in Yb^3+^doped Cs_2_NaSb_*x*_Bi_1–*x*_Cl_6_ NCs.

### Er^3+^ and Nd^3+^-Doped Cs_2_NaSb_*x*_Bi_1–*x*_Cl_6_ NCs

**Figure 5:**
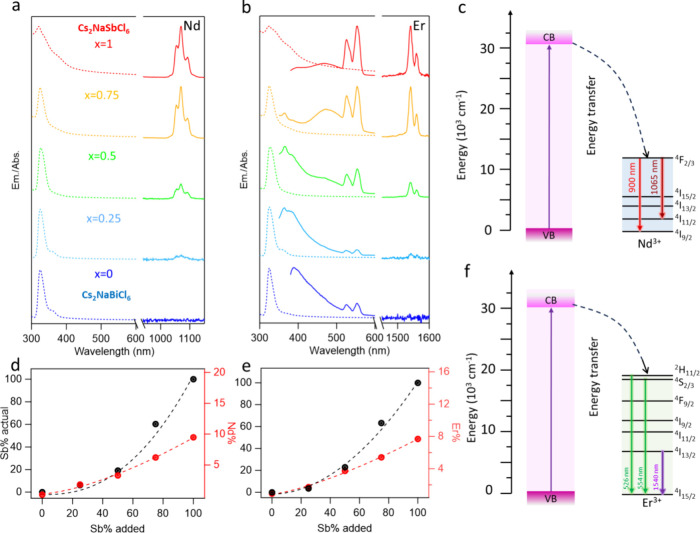
Normalized Absorption and NIR PL emissions of (a) Nd^3+^-doped Cs_2_NaSb_*x*_Bi_1–*x*_Cl_6_ NCs and (b) Er^3+^-doped Cs_2_NaSb_*x*_Bi_1–*x*_Cl_6_ NCs. Actual concentration by mol ratio of Sb^3+^ and Ln^3+^ of in (d) Nd^3+^-doped Cs_2_NaSb_*x*_Bi_1–*x*_Cl_6_ NCs and (e) Er^3+^-doped Cs_2_NaSb_*x*_Bi_1–*x*_Cl_6_ NCs by ICP measurements. Band alignment and electronic state energy levels for the (c) Nd^3+^ and (f) Er^3+^-doped Cs_2_NaSb_*x*_Bi_1–*x*_Cl_6_ NCs.

To further prove efficient Ln^3+^ doping into smaller Sb^3+^ substitutional sites, other lanthanide dopants, including Er^3+^ (103 pm) and Nd^3+^ (112 pm), with even larger ionic radius and polarizability, were doped in Sb^3+^/Bi^3+^-based Cs_2_NaSb_*x*_Bi_1–*x*_Cl_6_ perovskite NCs. Er^3+^ and Nd^3+^ dopant ions were chosen as they are widely known to effectively modify the optical properties of various NCs.^47−50,52,53,81,82^Figure S8a and S9a show the XRD patterns of Nd^3+^ doped Cs_2_NaSb_*x*_Bi_1–*x*_Cl_6_ NCs and Er^3+^ doped Cs_2_NaSb_*x*_Bi_1–*x*_Cl_6_ NCs, respectively. The peaks gradually shifted to higher angles as more Sb^3+^ ions were alloyed due to lattice shrinkage (Figure S8b and S9b). The absorption and NIR PL spectra of Nd^3+^ doped Cs_2_NaSb_*x*_Bi_1–*x*_Cl_6_ NCs is shown in Figure 5a with a 1065 nm NIR PL corresponding to the ^4^F_2/3_-^4^I_11/2_ electronic transition in the [NdCl_6_]^3–^ octahedra (Figure 5c).^82^ Er^3+^ doped Cs_2_NaSb_*x*_Bi_1–*x*_Cl_6_ NCs have a PL in both the visible and NIR ranges (Figure 5b). The green emission located at 526 and 554 nm originates from the ^2^H_11/2_–^4^I_15/2_ and ^4^S_3/2_–^4^I_15/2_ transitions of Er^3+^ f-electrons,^34,83^ respectively, while the strong NIR emission at ∼ 1541 nm in the NIR range is attributed to the ^4^I_13/2_ – ^4^I_15/2_ transition (Figure 5f). The NIR PL intensity and PL lifetime (Figure S8c, d and S9c, d) of both Nd^3+^ and Er^3+^ experience exponential growth by adding more Sb^3+^. The PL QY in NIR range of Nd^3+^ and Er^3+^ doped Cs_2_NaSbCl_6_ NCs are 4.2% and 1.7%, respectively (Table S1). Additionally, the ICP-OES data further confirms that the actual content of Sb^3+^ and Ln^3+^ dopants (Nd^3+^ and Er^3+^) both exhibit exponential growth upon the linear addition of Sb^3+^. These results suggest that the Sb-rich host can efficiently accommodate Ln^3+^ dopants and as more Ln^3+^ dopants are incorporated (Figure 5d, e), the PL of the Ln^3+^ dopants become more intense. Like Yb^3+^ dopants (101 pm), the ionic radius of Nd^3+^ (112 pm) and Er^3+^ (103 pm) ions are between that of Sb^3+^ (90 pm) and Bi^3+^ (117 pm). It should be noted that the ionic radius of Nd^3+^ is very close to that of Bi^3+^ with a very small cationic size mismatch of (∼4%). However, similar to Yb^3+^ and Er^3+^, Nd^3+^ ions also prefer to occupy the smaller O_h_ [SbCl_6_]^3–^ sites, which could be due to a higher polarizability of Nd^3+^ (3.900 Å^3^) than Er^3+^ (2.990 Å^3^) and Yb^3+^ (2.630 Å^3^).^63^

### Ln^3+^ (Yb^3+^, Er^3+^, and Nd^3+^)-Doped Vacancy-Induced Perovskite Cs_3_Sb_2*x*_Bi_2–2x_Cl_9_ and Layered Double Perovskite Cs_4_MnSb_2*x*_Bi_2–2x_Cl_12_ NCs

In order to prove that the higher doping efficiency of the lanthanide dopants is due to the smaller substitutional sites and not due to a specific perovskite crystal structure, we expand the type of Bi^3+^/Sb^3+^-based lead-free perovskites to vacancy-induced Cs_3_Sb_2*x*_Bi_2–2x_Cl_9_ and layered double perovskite Cs_4_MnSb_2*x*_Bi_2–2x_Cl_12_. Sb^3+^/Bi^3+^ based perovskites such as Cs_3_Bi_2_X_9_^22^ and Cs_4_MnSb_2_Cl_12,_^49,66,84^ which also have received attention due to their excellent stability, low-toxicity, high absorption cross-section and intense self-trapped exciton (STE) emission.^85^

In this work, we synthesized Yb^3+^ doped Cs_3_Sb_2*x*_Bi_2–2x_Cl_9_ and Cs_4_MnSb_2*x*_Bi_2–2x_Cl_12_ NCs by a hot injection method (see synthetic details in Experimental section). Figure S10a and S11a show the XRD pattern of Yb^3+^ doped Cs_4_MnSb_2*x*_Bi_2–2x_Cl_12_ NCs and Cs_3_Sb_2*x*_Bi_2–2x_Cl_9_ NCs. Cs_4_MnSb_2*x*_Bi_2–2x_Cl_12_ NCs show a trigonal phase with an *R*3̅*m* space group.^28,66^ The XRD patterns are consistent with their standards, indicating that alloying with Sb^3+^ does not either change the crystal structure or induce phase changes in Cs_4_MnBi_2_Cl_12_ NCs. For the Yb^3+^ doped Cs_3_Sb_*x*_Bi_1–*x*_Cl_9_ NCs, there is a phase change from orthorhombic to trigonal upon the addition of Sb^3+^, which is consistent the previous report of an orthorhombic unit cell with a *Pmcn* space group for Cs_3_Bi_2_Cl_9_ (Figure 1b)^64^ and trigonal phase with a *P*3̅*m*1 space group for Cs_3_Sb_2_Cl_9_ (Figure 1c, Figure S11).^86^ A previous study observed a gradual phase change from 0D Cs_3_BiCl_6_ to 3D Cs_2_AgBiCl_6_ and 2D Cs_4_MnBi_2_Cl_12_ by a postsynthetic cation addition method,^87^ which shows that it is possible to form a solid solution between the materials with different phases.

**Figure 6:**
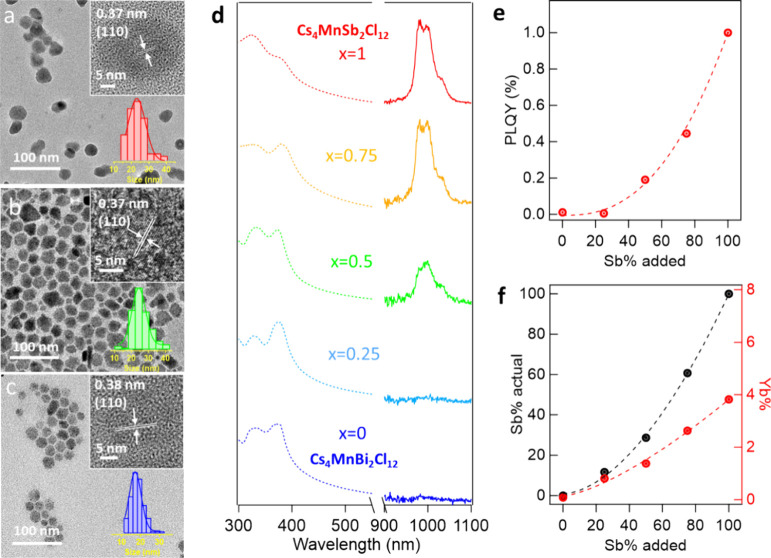
TEM images of (a) Yb^3+^-doped Cs_4_MnSb_2_Cl_12_ NCs, (b) Yb^3+^-doped Cs_4_MnSbBiCl_12_ NCs and (c) Yb^3+^-doped Cs_4_MnBi_2_Cl_12_ NCs, with the insets being respective size distribution histogram and high-resolution TEM images. (d) Normalized absorption and NIR PL for Cs_4_MnSb_2*x*_Bi_2–2x_Cl_12_ NCs. (e) NIR PL QYs vs Sb% concentration added in the Yb^3+^-doped Cs_4_MnSb_2*x*_Bi_2–2x_Cl_12_ NCs. (f) Actual concentration of Sb^3+^ and Yb^3+^ in Yb^3+^-doped Cs_4_MnSb_2*x*_Bi_2–2x_Cl_12_ NCs by ICP measurements vs Sb% concentration added.

**Figure 7:**
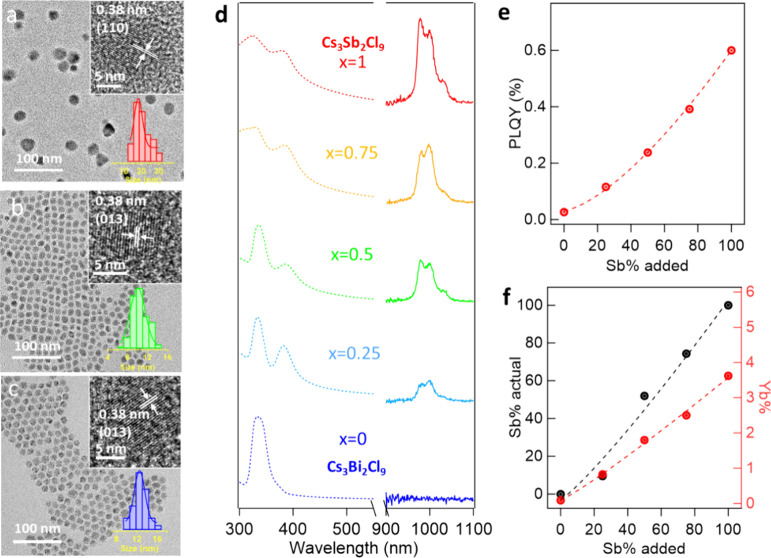
TEM images of (a) Yb^3+^-doped Cs_3_Sb_2_Cl_9_ NCs, (b) Yb^3+^-doped Cs_3_SbBiCl_9_ NCs, and (c) Yb^3+^-doped Cs_3_Bi_2_Cl_9_ NCs, with the insets being respective size distribution histogram and high-resolution TEM images. (d) Normalized absorption and NIR PL emission for Cs_3_Sb_2*x*_Bi_2–2x_Cl_9_ NCs. (e) NIR PL QYs vs Sb% concentration added in the Yb^3+^-doped Cs_3_Sb_2*x*_Bi_2–2x_Cl_9_ NCs. (f) Actual concentration of Sb^3+^ and Yb^3+^ in Yb^3+^-doped Cs_3_Sb_2*x*_Bi_2–2x_Cl_9_ NCs by ICP measurements vs Sb% concentration added.

The TEM images of Yb^3+^ doped Cs_4_MnSb_2*x*_Bi_2–2x_Cl_12_ NCs are shown in Figure 6a-c. The sizes of x = 0, 0.5, and 1, samples are 18.4 ± 3.5 nm, 25.7 ± 5.0 nm and 24.2 ± 6.0 nm, respectively. The high-resolution TEM images of Yb^3+^ doped Cs_4_MnSb_2*x*_Bi_2–2x_Cl_12_ NCs show a clear lattice corresponding to (110) lattice planes, which expands from 0.37 to 0.38 nm with increased Bi^3+^ concentration (insets of Figure 6a-c). The corresponding FFT images are shown in Figure S3 g-l. Highly monodispersed morphologies were observed with sizes of 12.6 ± 1.4 nm, 10.3 ± 1.5 and 19.9 nm ± 4.3 nm for Cs_3_Bi_2_Cl_9_, Cs_3_SbBiCl_9_, and Cs_3_Sb_2_Cl_9_ NCs, respectively (Figure 7a-c). A *d*-spacing of 0.38 nm was observed in Cs_3_Bi_2_Cl_9_ NCs, which is assigned to the (013) plane (inset of Figure 7c). In addition, a 0.38 nm *d*-spacing of (110) plane Cs_3_Sb_2_Cl_9_ NCs was observed (inset of Figure 7a). The corresponding FFT of the High-resolution TEM images are shown in Figure S3 m-r. The lattice fringes are expected to be bigger for NCs with higher Bi^3+^ concentration under the same crystal structure due to the larger ionic radius of Bi^3+^. However, in the case of Cs_3_Sb_2*x*_Bi_2–2x_Cl_9_ NCs, the crystal system changes from trigonal to orthorhombic, which cancels out the size changes caused by the larger ion addition, resulting in no obvious change in the lattice fringes observed. These results are consistent with the relatively small peak shift observed in the XRD from Yb^3+^ doped Cs_3_Bi_2_Cl_9_ to Yb^3+^ doped Cs_3_Sb_2_Cl_9_ NCs (Figure S11b).

At low Sb content in the host NCs (*x* < 0.5), Yb^3+^ doped Cs_4_MnSb_2*x*_Bi_2–2x_Cl_12_ NCs and Cs_3_Sb_2*x*_Bi_2–2x_Cl_9_ NCs show negligible emission from Yb^3+^ at ∼ 990 nm, while the emission increases dramatically when *x* ≥ 0.5 (Figure 6d-e, 7d-e). The PL QY in the NIR range of Yb^3+^ doped Cs_4_MnSb_2_Cl_12_ NCs and Cs_3_Sb_2_Cl_9_ NCs are 1.0% and 0.6%, respectively (Table S1). ICP-OES data indicates that the actual concentration of Sb^3+^ and Yb^3+^ are extremely low in the range of *x* < 0.5 and experience an exponential growth when *x* ≥ 0.5 (Figure 6f, 7f). A similar trend is found in Yb^3+^ doped Cs_4_MnSb_2*x*_Bi_2–2x_Cl_12_ NCs and Cs_3_Sb_2*x*_Bi_2–2x_Cl_9_ NCs to the Yb^3+^ doped Cs_2_NaSb_*x*_Bi_1–*x*_Cl_6_ NCs, which suggests that Sb^3+^ could function as an excellent host site for efficient lanthanide doping in not only double perovskite Cs_2_NaSb_*x*_Bi_1–*x*_Cl_6_, but also layered double perovskite Cs_4_MnSb_2*x*_Bi_2–2x_Cl_12_ and vacancy-induced perovskite Cs_3_Sb_2*x*_Bi_2–2x_Cl_9_.

It should be noted that there are two B sites (Mn^2+^ and Sb^3+^) with different oxidation states in Cs_4_MnSb_2_Cl_12_ NCs. To prove isovalent substitutional doping of Ln^3+^ into Sb^3+^ sites, we synthesized Yb^3+^ doped Cs_4_Cd_*x*_Mn_1–*x*_Sb_2_Cl_12_ NCs to study the effect of the B(II) site on the doping efficiency of Yb^3+^ (Figure S12). Based on ICP results, the content of the Yb^3+^ almost stayed the same upon the addition of Cd^2+^ (Figure S12e), indicating that the [B^(II)^Cl_6_]^4–^ octahedra are not a preferred site of Yb^3+^ dopants and have negligible influence on the doping efficiency of Yb^3+^ ions. The NIR emission is the most intense when x is 0.5 in Cs_4_Mn_0.5_Cd_0.5_Sb_2_Cl_12_ NCs (Figure S12a, d). It is reported that Mn^2+^ ions are an “energy transfer shuttle” in Ln doped Cs_4_MnSb_2_Cl_12_ crystals, and the energy path way is host → Mn^2+^ → Ln^3+^ energy transfer.^49^ Therefore, the NIR emission is not as intense when *x* > 0.5 due to the concentration quenching effect between Mn–Mn magnetic coupling pairs.^88^

Er^3+^ and Nd^3+^ doped Cs_4_MnSb_2*x*_Bi_2–2x_Cl_12_ NCs were also synthesized (Figure S13 and S14). Interestingly, the ∼ 670 nm red emission of Er^3+^ in Cs_4_MnSb_2*x*_Bi_2–2x_Cl_12_ NCs is not green as in the Er^3+^ doped Cs_2_NaSbCl_6_ (∼ 558 and 585 nm), which can be assigned to the ^4^F_9/2_-^4^I_15/2_ transition. The reason is that the bandgap of Cs_2_NaSbCl_6_ is large enough (∼3.2 eV) to excite the Er^3+^ to the ^4^S_2/3_ state and the energy is directly transferred from the Cs_2_NaSbCl_6_ host to Er^3+^ dopant ions. Then a ^4^S_2/3_-^4^I_15/2_ transition occurs and green light is emitted. However, for Er^3+^ doped Cs_4_MnSb_2*x*_Bi_2–2x_Cl_12_ NCs, energy is transferred through the host → Mn^2+^ → Er^3+^ pathway (Figure S15).^49^ Our optical data also supports the host → Mn^2+^ → Er^3+^ pathway as when more Sb^3+^ is incorporated, the emission of Er^3+^ increases while the emission from Mn^2+^ decreases, indicating the energy transfer from Mn^2+^ to Er^3+^. The energy difference between the ground state (^6^A_1_) and excited state (^4^T_1_) of Mn^2+^ is ∼ 2.1 eV, which is too small to excite Er^3+^ ions to ^4^S_2/3_ state, instead, the Er^3+^ dopants would be excited to the ^4^F_9/2_ state, and the ^4^F_9/2_-^4^F_15/2_ transition emits orange light at ∼ 670 nm. Notably, regardless of the different energy transfer pathway(s) in the host Cs_4_MnSb_2*x*_Bi_2–2x_Cl_12_ perovskite NCs, the Er^3+^ and Nd^3+^ PL increases with the addition of Sb^3+^ ions in the host materials (Figure S13a, d and S14a, d). ICP results confirm that the increase of the PL is due to the increasing doping efficiency of the Er^3+^ and Nd^3+^ dopants (Figure S13e and S14e). Those results further illustrate that the design of suitable doping sites for enhanced Ln^3+^ doping in lead-free perovskite is highly effective and efficient.

### Environmental Stability

Stability tests were conducted on Cs_2_NaSbCl_6_ NCs doped with different Ln^3+^ ions, Yb^3+^-doped Cs_3_Sb_2_Cl_9_ NCs, and Yb^3+^-doped Cs_4_MnSb_2_Cl_12_ NCs (Figures S16–18). The results indicate that double perovskite NCs demonstrate superior stability compared to layered double perovskite NCs and vacancy-induced perovskite NCs. In the heating tests, all three Yb^3+^-doped perovskite NCs exhibited PL enhancement during the first few cycles, likely due to thermal annealing increasing the crystallinity of the NCs.^89,90^ For the Yb^3+^-doped double perovskite NCs, the PL remained at 44% of the original intensity after 17 cycles of heating, while the PL of Yb^3+^-doped vacancy-induced and layered double perovskite NCs was reduced to 42% and 35%, after 7 and 11 cycles, respectively (Figure S16 a-d). The light stability of double perovskite NCs also showed that Yb^3+^-doped double perovskite NCs were more stable compared to the other two perovskites: after 2 h of blue light irradiation, Yb^3+^-doped Cs_2_NaSbCl_6_ retained 88% of the original NIR PL. However, for the Yb^3+^-doped Cs_3_Sb_2_Cl_9_ and Cs_4_MnSb_2_Cl_12_ NCs, the PL was reduced to 53% and 20%, respectively (Figure S17**a-d**). After being stored in ambient conditions for 40 days, the NIR emissions of Yb^3+^-doped Cs_2_NaSbCl_6_, Cs_3_Sb_2_Cl_9_, and Cs_4_MnSb_2_Cl_12_ NCs decreased to 57%, 32%, and 28% of the initial intensities, respectively (Figure S18**a-d**). The relatively low stability of layered double perovskites and vacancy-induced perovskites might be attributed to intrinsic vacancies, which introduce more surface defects, making them more prone to oxidation and reactions with polar solvents. The double perovskite is superior host to other two types of lead-free perovskites based on the environmental stability and PLQY (Figure S16-18,Table S1).

In addition, the type of Ln^3+^ dopant has minimal impact on the stability of the perovskite NCs. Yb^3+^, Nd^3+^, and Er^3+^-doped perovskite NCs showed similar PL enhancements after being heated for a few cycles, and they retained ∼ 30% of the original PL after 17 cycles of heating/cooling (Figure S16 e-h). Furthermore, they displayed similar light (Figure S17 e-h) and ambient stabilities (Figure S18 e-h). These results are likely due to the low concentration of dopants in the host NCs, with negligible impact of dopant type on the stability of the host materials.

### Computational Calculations

**Figure 8:**
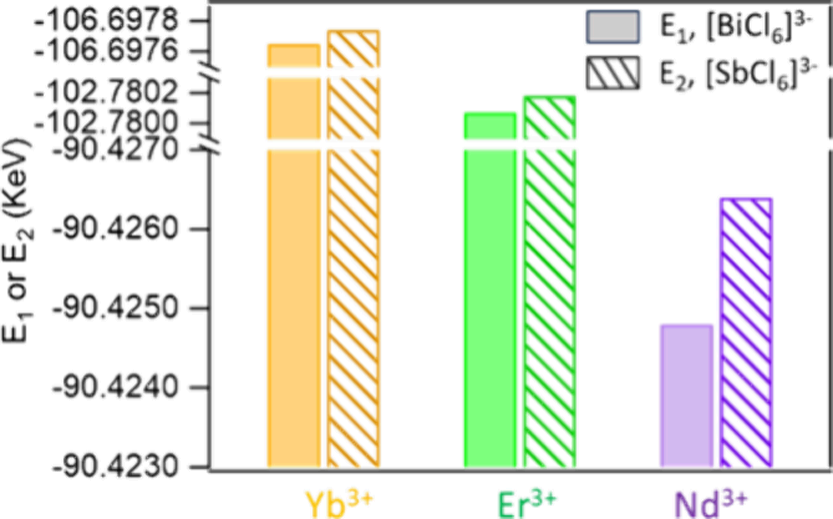
Relative energy calculations for Ln^3+^ ions in [SbCl_6_]^3–^ and [BiCl_6_]^3–^ cages at 0 K with bond lengths of 2.683 and 2.647 Å, respectively.

To investigate the preference of Ln^3+^ substitutions in [SbCl_6_]^3–^ over [BiCl_6_]^3–^ O_h_ sites, we performed computational calculations to determine the relative energies of Ln^3+^ ions in both substitutional environments. Density Functional Theory (DFT) calculations were carried out using the B3LYP functional and def2-TZVP basis set with Effective Core Potentials (ECP) for the lanthanide ions. These calculations were performed with the Psi4 computational package.^91,92^ The octahedral voids of [SbCl_6_]^3–^ and [BiCl_6_]^3–^ systems were constructed based on the bond lengths reported in the literature, which are 2.647 Å and 2.683 Å, respectively.^75^ The DFT calculations revealed that all the Ln^3+^ ions (Nd^3+^, Er^3+^, and Yb^3+^) exhibit a preference for substitution in [SbCl_6_]^3–^ octahedra over [BiCl_6_]^3–^ octahedra. The difference in formation energy of replacing Bi^3+^ (eq 1) and Sb^3+^ (eq 2) by Ln^3+^ in [B(III)Cl_6_]^3–^ octahedron is defined as E_1_ and E_2_, respectively. E_2_ is lower than E_1_ for all three Ln^3+^ dopant ions, with the difference between E_1_ and E_2_ for Yb^3+^, Er^3+^ and Nd^3+^ being 0.0900, 0.110, and 1.55 eV, respectively (Figure 8 and Table S2). These results indicate that the Ln^3+^ substituted [SbCl_6_]^3–^ octahedra are more stable compared to Ln^3+^ substituted [BiCl_6_]^3–^ octahedra. The simulation results are consistent with higher doping efficiency observed in Ln^3+^ doped Sb^3+^-based perovskite NCs, as evidenced by experimental data including XRD, optical data, and ICP-OES elemental analysis.
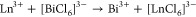
1
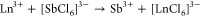
2

## Conclusions

To study the doping site-dependent Ln^3+^ doping in lead-free perovskite NCs, a series of Ln^3+^ (Yb^3+^, Er^3+^ and Nd^3+^) doped Sb^3+^/Bi^3+^-based lead-free perovskite NCs, including double perovskite Cs_2_NaSb_*x*_Bi_1–*x*_Cl_6_, vacancy-induced perovskite Cs_3_Sb_2*x*_Bi_2–2x_Cl_9_ and layered double perovskite Cs_4_MnSb_2*x*_Bi_2–2x_Cl_12_ NCs have been developed and synthesized. NIR emissions were observed in Ln^3+^ doped lead-free perovskites due to the f-f transition in the incorporated Ln^3+^ ions. For all Ln^3+^ doped perovskite NCs with different dopant PL behaviors resulting from varying host-to-dopant energy transfer mechanisms in the three lead-free perovskite NCs, it was found that the Ln^3+^ NIR emission and the actual Ln^3+^ doping concentration increase as more Sb^3+^ is incorporated into the Sb^3+^/Bi^3+^-alloyed perovskite NCs, despite the considerable size mismatch between Sb^3+^ and Ln^3+^. More significantly, one of the largest lanthanide ions, Nd^3+^, still shows higher doping efficiency in smaller Sb^3+^-based perovskite NCs. We attribute this result to the relatively high polarizability of Ln^3+^ ions, which assists the Ln^3+^ to fit into the smaller host substitutional B(III) sites to make a more structurally stable doped NCs. The stronger second-order Jahn–Teller distortion in [SbCl_6_]^3–^ octahedra might also contribute to the higher doping efficiency. Our work provides a deeper understanding of the behavior of Ln^3+^ dopant and basic guidance for future development of novel Ln^3+^ doped perovskite NCs.

## Experimental Section

### Chemicals

Cesium acetate (Cs(OAc), 99.9%, Alfa Aesar), bismuth acetate (Bi(OAc)_3_, 99.999%, Alfa Aesar), antimony acetate (Sb(OAc)_3_, 97%, Alfa Aesar), anhydrous manganese acetate (Mn(OAc)_2_, 98%, Alfa Aesar), cadmium acetate (Cd(OAc)_2_, 99.99%, Sigma-Aldrich), sodium acetate (Na(OAC), 99.99%), ytterbium acetate hydrate (Yb(OAc)_3_, 99.9%, Thermo Scientific), erbium acetate tetrahydrate (Er(OAc)_3_·4H_2_O, 99.9%, Thermo Scientific), neodymium acetate hydrate (Nd(OAc)_3_, 99.9%, Thermo Scientific), ytterbium(III) chloride hexahydrate (YbCl_3_·6H_2_O, 99.9%, Simga Aldrich), dimethylformamide (DMF, 99.9%, Fisher), chlorotrimethylsilane (TMS-Cl, 99%, Sigma-Aldrich), 1-octadecene (ODE, 90%, Thermo Scientific), oleic acid (OA, 90%, Thermo Scientific), oleylamine (OAm, 70%, Sigma-Aldrich), toluene (HPLC-UV grade, Pharmco), *n*-hexane (HPLC-UV grade, Pharmco), and ethyl acetate (HPLC-UV grade, Pharmco).

### Synthesis of Ln^3+^-Doped Cs_2_NaSb_*x*_Bi_1–*x*_Cl_6_ NCs (*x* = 0, 0.25, 0.5, 0.75, and 1)

The Ln^3+^ doped Cs_2_NaSb_*x*_Bi_1–*x*_Cl_6_ NCs were synthesized by following a previously reported hot-injection method with slight modification.^65^ Briefly, Cs(OAc) (68 mg, 0.35 mmol), Na(OAc) (21 mg, 0.25 mmol), Bi(OAc)_3_ (96 × (1-*x*) mg, 0.25 × (1-*x*) mmol), Sb(OAc)_3_ (75 × *x* mg, 0.25 × *x* mmol), and 20% Ln dopant precursor Ln(OAc)_3_ (0.05 mmol) were mixed with 5 mL ODE, 1.5 mL OA and 0.3 mL OAm in a three-neck flask. The mixture was heated to 110 °C under vacuum for 1 h to remove oxygen and water, and further heated to 200 °C under Argon. 0.2 mL TMS-Cl was swiftly injected into the mixture. Upon reaction for ∼ 5 s, the mixture was cooled to room temperature using a water bath. The as-synthesized NCs were collected by centrifuging at 5000 rpm for 5 min. The precipitate was redissolved in toluene for further characterization.

### Synthesis of Ln^3+^-Doped Cs_3_Sb_2*x*_Bi_2(1–*x*)_Cl_9_ NCs (*x* = 0, 0.25, 0.5, 0.75, and 1)

The Ln^3+^ doped Cs_3_Sb_2*x*_Bi_2(1-x)_Cl_9_ NCs were synthesized by following a previously reported method with slight modification.^64^ Typically, Cs(OAc) (68 mg, 0.35 mmol), Mn(OAc)_2_ (22 mg, 0.125 mmol), Bi(OAc)_3_ (96 × (1-*x*) mg, 0.25 × (1-*x*) mmol), Sb(OAc)_3_ (75 × *x* mg, 0.25 × *x* mmol), and Ln(OAc)_3_ (0.05 mmol) were mixed with 5 mL ODE, 1.5 mL OA and 0.3 mL OAm in a three-neck flask. The mixture was heated to 110 °C under vacuum for 1 h to remove oxygen and water, and further heated to 170 °C under Argon flow. 0.2 mL TMS-Cl was swiftly injected into the mixture. Upon reaction for 5 min, the mixture was cooled to room temperature using a water bath. The as-synthesized NCs were collected by centrifuging at 5000 rpm for 5 min. The precipitate was redissolved in toluene for further characterization.

### Synthesis of Ln^3+^-Doped Cs_4_MnSb_2*x*_Bi_2(1–*x*)_Cl_12_ NCs (*x* = 0, 0.25, 0.5, 0.75, and 1)

The Ln^3+^ doped Cs_4_MnSb_2*x*_Bi_2(1-x)_Cl_12_ NCs were synthesized by following a previously reported method with slight modification.^66^ Typically, Cs(OAc) (68 mg, 0.35 mmol), Mn(OAc)_2_ (22 mg, 0.125 mmol), Bi(OAc)_3_ (96 × (1-*x*) mg, 0.25 × (1-*x*) mmol), Sb(OAc)_3_ (75 × *x* mg, 0.25 × *x* mmol), and Ln(OAc)_3_ (0.05 mmol) were mixed with 5 mL ODE, 1.5 mL OA and 0.3 mL OAm in a three-neck flask. The mixture was heated to 110 °C under vacuum for 1 h to remove oxygen and water, and further heated to 170 °C under Argon flow. 0.2 mL TMS-Cl was swiftly injected into the mixture. Upon reacting for 1 min, the mixture was cooled to room temperature using a water bath. The as-synthesized NCs were collected by centrifuging at 5000 rpm for 5 min. The precipitate was redissolved in toluene for further characterization.

### Characterization

Powder X-ray diffraction (XRD) patterns were taken on a Bruker D2 Phaser with a LYKXEYE 1D silicon strip detector using Cu Kα radiation (λ = 1.5406 Å). Transmission electron microscopy (TEM) images were obtained on JEM 2100F (operated at an accelerating voltage of 200 kV). The UV–vis absorption measurements were collected on an Agilent Cary 60 spectrophotometer. The photoluminescence (PL) measurements were performed with a Horiba FluoroMax Plus spectrofluorometer. Time-resolved emission measurements were conducted using an Edinburgh FLS-980 spectrofluorometer with a photomultiplier tube (PMT, R928 Hamamatsu) detector. For Ln^3+^ emission lifetime measurements, the pulsed excitation light was generated by an μF2 60 W xenon flashlamp operating at a repetition rate of 100 Hz. ICP-OES analysis was performed on a PerkinElmer Avio 220. 2% of nitric acid was used to digest the NC samples.

## Supporting Information Available

Additional experimental details, XRD, optical data, and TEM image of Ln^3+^-doped perovskite NCs synthesized through direct method and cation exchange method (PDF)
