# Supplementary material for: Tailoring Substitutional Sites for Efficient Lanthanide Doping in Lead-Free Perovskite Nanocrystals with Enhanced Near-Infrared Photoluminescence
Source: ACS Nano. 2025 Apr 11;19(15):14941–53. doi: 10.1021/acsnano.5c00487 (PMC12020422; doi:10.1021/acsnano.5c00487)
Supplement: Supplementary file 1 — nn5c00487_si_001.pdf [file nn5c00487_si_001.pdf]

Supporting information for

Tailoring Substitutional Sites for Efficient Lanthanide Doping in Lead-Free Perovskite Nanocrystals with Enhanced Near-Infrared Photoluminescence

*Hanjie Lin, Sara Talebi, Walker MacSwain, Vanshika Vanshika, Arindam Chakraborty, and Weiwei Zheng**

Department of Chemistry, Syracuse University, Syracuse, New York 13244, United States

Table of contents

Experimental

Synthesis of undoped $Cs_2NaSb_xBi_{1-x}Cl_6$ NCs.

Synthesis of Yb^{3+} doped $Cs_2NaSb_xBi_{1-x}Cl_6$ NCs with different doping concentrations.

Cation exchange for doping Yb^{3+} in $Cs_2NaSb_xBi_{1-x}Cl_6$ NCs.

Stability tests.

PL QY measurement.

Figures

Figure S1. NIR PL intensity of Yb^{3+} doped $Cs_2NaSbCl_6$ NCs with different doping concentrations.

Figure S2. XRD pattern of undoped $Cs_2NaSb_xBi_{1-x}Cl_6$ NCs.

Figure S3. High-resolution TEM images and corresponding FFT doped NCs.

Figure S4. Average B^{3+} ionic radius and lattice mismatch in Yb^{3+} doped $Cs_2NaSb_xBi_{1-x}Cl_6$ NCs.

Figure S5. Optical data and elemental analysis of Yb^{3+} doped $Cs_2NaSbCl_6$ NCs through cation exchange doping method.

Figure S6. Optical data and elemental analysis of Yb^{3+} doped $Cs_2NaSbCl_6$ NCs synthesized by Bz-Cl.

Figure S7. TEM image, XRD pattern, and optical data of Yb^{3+} doped $Cs_2NaBiCl_6$ NCs synthesized at 200 °C.

Figure S8. XRD patterns and PL life time of Nd^{3+} doped $Cs_2NaSb_xBi_{1-x}Cl_6$ NCs.

Figure S9. XRD patterns and PL life time of Er^{3+} doped $Cs_2NaSb_xBi_{1-x}Cl_6$ NCs.

Figure S10. XRD pattern of Yb^{3+} doped $Cs_4MnSb_{2x}Bi_{2-2x}Cl_{12}$ NCs.

Figure S11. XRD pattern of Yb^{3+} doped $Cs_3Sb_{2x}Bi_{2-2x}Cl_9$ NCs.

Figure S12. XRD patterns, optical properties, and ICP results of Yb^{3+} doped $Cs_4Mn_{1-x}Cd_xSb_2Cl_{12}$ NCs.

Figure S13. XRD patterns, optical properties, and ICP results of Er^{3+} doped $Cs_4MnSb_{2x}Bi_{2-2x}Cl_{12}$ NCs.

Figure S14. XRD patterns, optical properties, and ICP results of Nd^{3+} doped $Cs_4MnSb_{2x}Bi_{2-2x}Cl_{12}$ NCs.

Figure S15. Scheme illustrations of energy level alignment and energy transfer pathway for Ln^{3+} doped $Cs_4MnSb_2Cl_{12}$ NCs.

Figure S16. Heat stability of doped lead-free perovskite NCs.

Figure S17. Light stability of doped lead-free perovskite NCs.

Figure S18. Air stability of doped lead-free perovskite NCs.

Table

Table S1. NIR PLQYs of doped lead-free perovskite NCs.

Table S2. DFT energy calculations for Ln^{3+} ions doped in $[SbCl_6]^{3-}$ and $[BiCl_6]^{3-}$ O_h sites.

Experimental

Synthesis of $Cs_2NaSb_xBi_{1-x}Cl_6$ NCs (x = 0, 0.25, 0.5, 0.75,* and *1). The $Cs_2NaSb_xBi_{1-x}Cl_6$ NCs were synthesized by following a previously reported hot-injection method with slight modifications.[1] Cs(OAc) (68 mg, 0.35 mmol), Na(OAc) (21 mg, 0.25 mmol), $Bi(OAc)_3$ (96 × (1-*x*) mg, 0.25 × (1-*x*) mmol), $Sb(OAc)_3$ (75 × *x* mg, 0.25 × *x* mmol), were mixed with 5 mL ODE, 1.5 mL OA and 0.3 mL OAm in a three-neck flask. The mixture was heated to 110 °C under vacuum for 1 h to remove oxygen and water, then further heated to 200 °C under Argon flow. 0.2 mL TMS-Cl was swiftly injected into the mixture. Upon reaction for ~5 s, the mixture was cooled to room temperature using a water bath. The as-synthesized NCs were collected by centrifuging at 5000 rpm for 5 min. The precipitate was redissolved in toluene for further characterization.

Synthesis of Yb^{3+} doped $Cs_2NaSb_xBi_{1-x}Cl_6$ NCs with different amount of Yb^{3+} precursors (x = 0, 0.25, 0.5, 0.75,* and *1). The procedures are the same as the synthesis of undoped $Cs_2NaSb_xBi_{1-x}Cl_6$ NCs, but 0.0125, 0.025, 0.05 and 0.075 mmol of $Yb(OAc)_3$ were added to the synthesis for the 5, 10, 20, and 30% doped samples.

Cation exchange. The $YbCl_3$ stock solution was prepared by dissovling 387 mg (1 mmol) of $YbCl_3 \cdot 6H_2O$ in 2 mL of DMF. Then, 60 mg NCs were dissolved in 3.5 mL of toluene. 0.25 mL of $YbCl_3$ stock solution was added to the NCs solution, and the mixture was stir under room temperature for 15 hours. The mixture was centrifuged under 5000 rpm for 5 min, the supernatant decanted, then the precipitate was redispersed in ~3 mL toluene, and the purfied NCs toluene solution was used for optical measurements. ~10 mL of ethyl acetate was added to crash the NCs out. The purification process was repeated 5 times for ICP-OES measurements.

Stability tests. For thermal stability tests, the NCs were deposited onto glass slides and heated to 200 °C on a hot plate for 10 minutes during each cycle. After 10 min heating, the samples were allowed to cool under ambient conditions for PL measurements. Additional thermal cycles were performed on the NCs to evaluate their thermal stability. For light stability tests, the NC samples were dispersed in toluene and exposed to blue LED irradiation (wavelength: 405 ± 15 nm) with a power intensity of 250 ± 40 mW cm^{-2}. Additionally, ambient/air stability tests were conducted to assess the NCs' resistance to environmental conditions at 20 °C and a relative humility (RH) of 70%.

PL QY measurement. In this measurement, Rhodamine 6G emits light in the visible range, while the samples emit NIR light. To compare the PL intensities between the reference and NC samples, a scaling factor was calculated. This scaling factor represents the ratio of the responsivity of the visible detector to that of the NIR detector. It was determined by aligning and connecting the spectra of the same IR-808 dye, collected separately using the visible and NIR signal detectors, under identical measurement conditions.[2] The equation used for the calculation of the PLQYs of the samples is:

$$\Phi_U = \Phi_R \left(\frac{A_R}{A_U}\right)\left(S\frac{E_U}{E_R}\right)\left(\frac{I_R}{I_U}\right)\left(\frac{\eta_U}{\eta_R}\right)$$

where S is the scaling factor, Φ is the PLQY, A is the absorption, E is the integrated fluorescence intensity of the emitted light, I is the intensity of the excitation light, and η is the index of refraction of the solvent for the solution. The subscript "U" denotes the material treated as the unknown, while the subscript "R" denotes the material treated as the known standard to which the unknown is being compared.

Table S1. PLQYs in NIR range of Ln^{3+}-doped lead-free perovskite NCs

Samples	NIR PLQY (%)
Yb^{3+} doped $Cs_2NaSbCl_6$ NCs	1.8%
Yb^{3+} doped $Cs_4MnSb_2Cl_{12}$ NCs	1.0%
Yb^{3+} doped $Cs_3Sb_2Cl_9$ NCs	0.6%
Nd^{3+} doped $Cs_2NaSbCl_6$ NCs	4.2%
Nd^{3+} doped $Cs_4MnSb_2Cl_{12}$ NCs	1.9%
Er^{3+} doped $Cs_2NaSbCl_6$ NCs	1.7%
Er^{3+} doped $Cs_4MnSb_2Cl_{12}$ NCs	1.6%

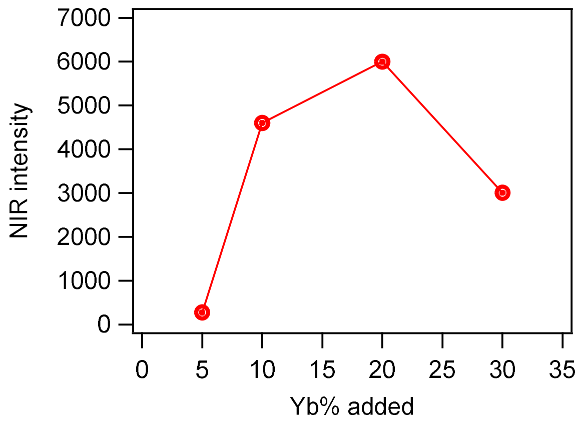

Figure S1. PL intensity at 990 nm of Yb^{3+} doped $Cs_2NaSbCl_6$ NCs with different doping concentrations.

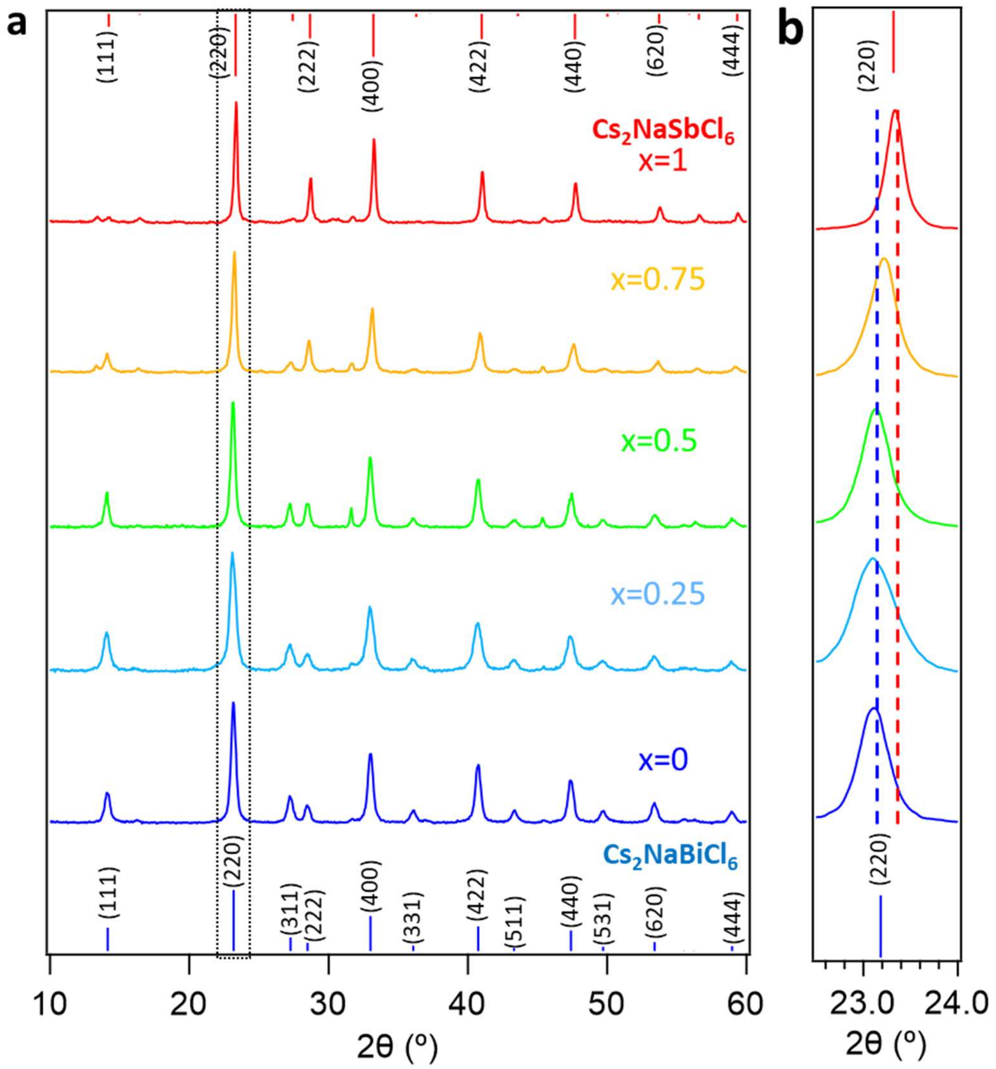

Figure S2. (a) XRD pattern of undoped $Cs_2NaSb_xBi_{1-x}Cl_6$ NCs. (b) Zoomed-in XRD patterns showing peak shifting of the (220) diffraction peak by alloying Sb^{3+} into $Cs_2NaBiCl_6$ NCs.

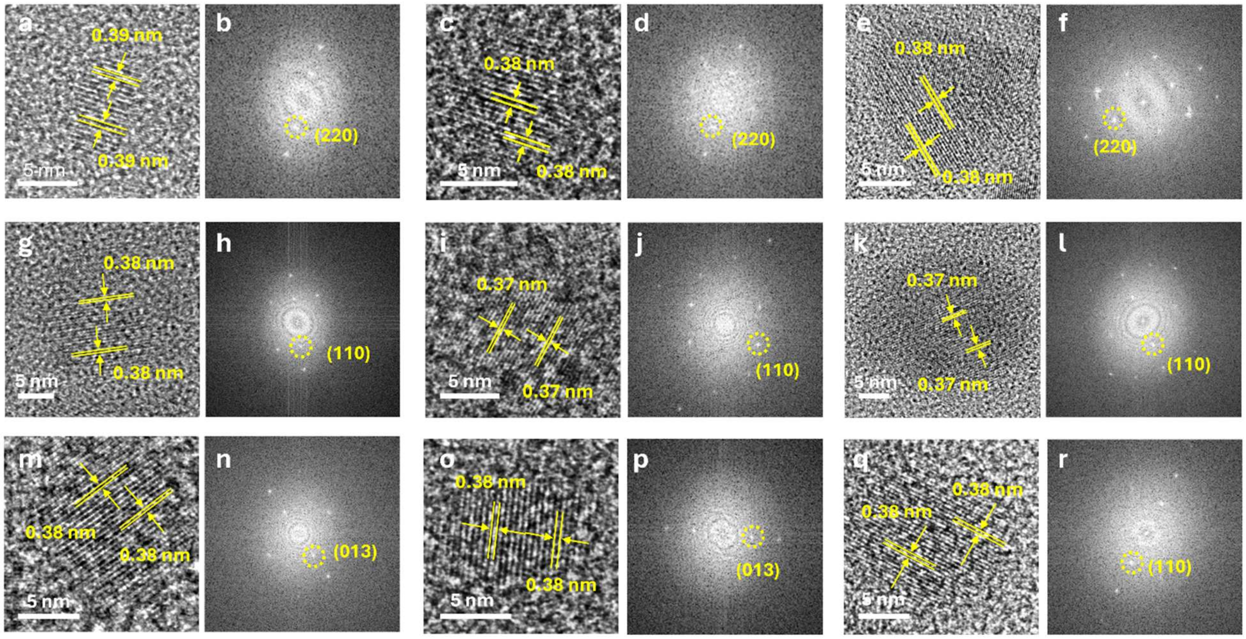

Figure S3. High-resolution TEM images and corresponding FFT of (a-b) $Cs_2NaBiCl_6$, (c-d) $Cs_2NaSb_{0.5}Bi_{0.5}Cl_6$, (e-f) $Cs_2NaSbCl_6$, (g-h) $Cs_4MnBi_2Cl_{12}$, (i-j) $Cs_4MnSbBiCl_{12}$, (k-l) $Cs_4MnSb_2Cl_{12}$, (m-n) $Cs_3Bi_2Cl_9$, (o-p) $Cs_3SbBiCl_9$, and (q-r) $Cs_3Sb_2Cl_9$ NCs.

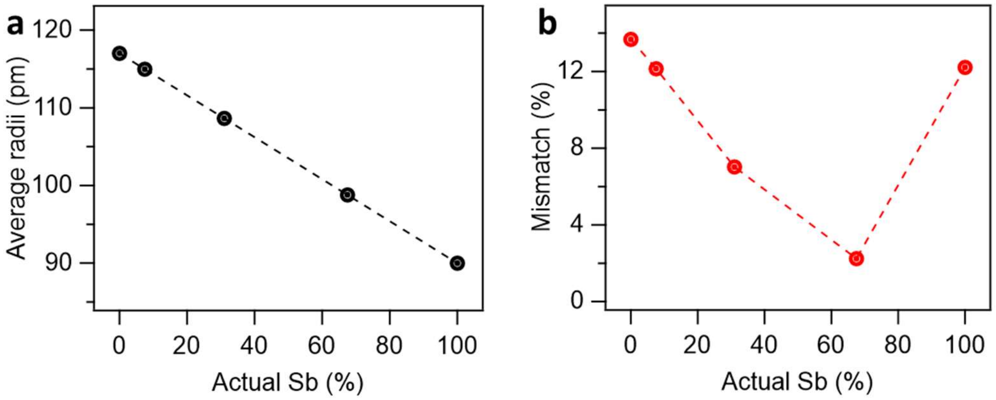

Figure S4. (a) Average B^{3+} ionic radius and (b) cationic radius mismatch between B^{3+} and Yb^{3+} in $Cs_2NaSb_xBi_{1-x}Cl_6$ NCs.

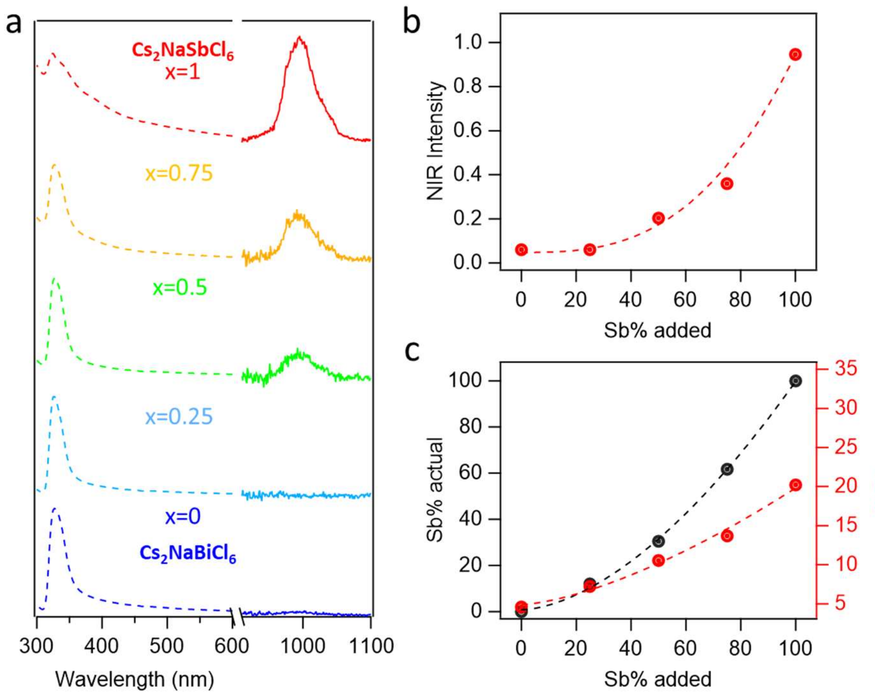

Figure S5. (a) Normalized absorption and NIR PL, (b) NIR PL intensities, and (c) ICP-OES elemental analysis results of Yb^{3+} doped $Cs_2NaSb_xBi_{1-x}Cl_6$ NCs through a cation exchange doping method.

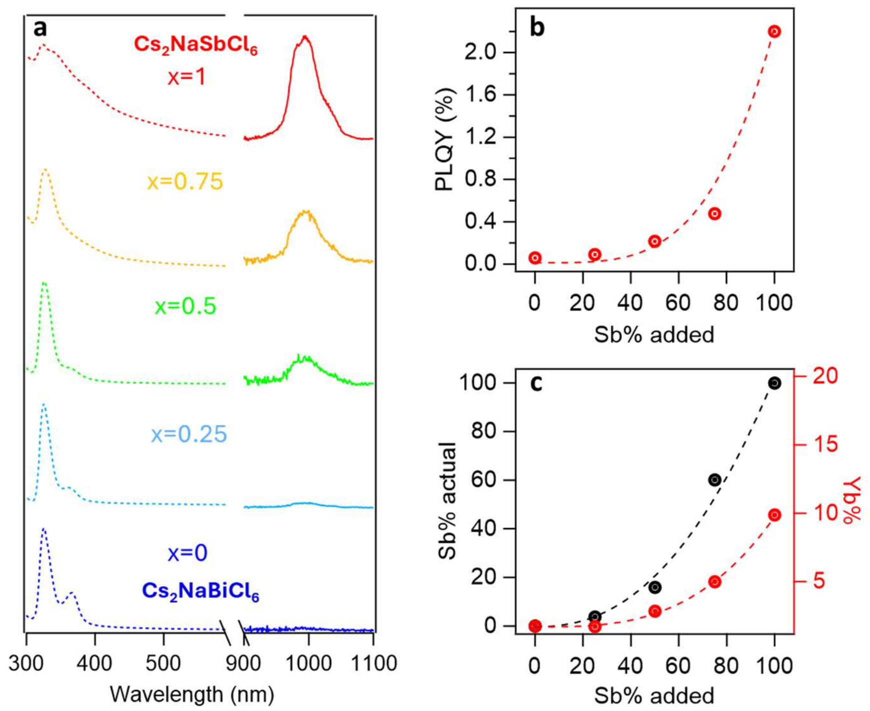

Figure S6. (a) Normalized absorption and NIR PL emission, (b) NIR PL QYs vs. Sb% concentration and (c) actual concentration of Sb^{3+} and Yb^{3+} by ICP measurements vs. Sb% concentration added of Yb^{3+} doped $Cs_2NaSb_xBi_{1-x}Cl_6$ NCs synthesized by benzoyl chloride.

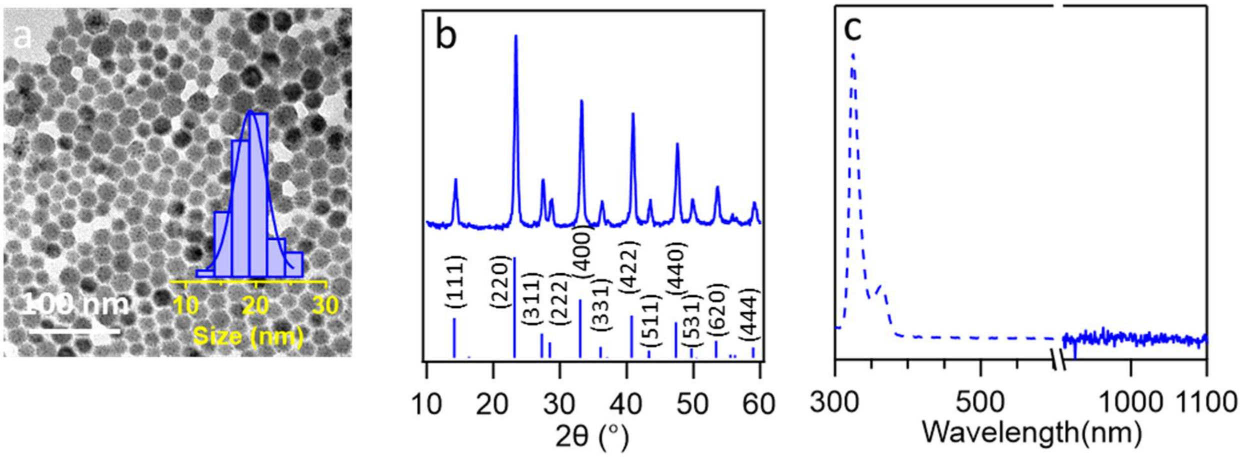

Figure S7. (a) TEM image, with inset histogram showing size distribution, (b) XRD pattern, (c) absorption (dash line) and PL (solid line) of Yb^{3+} doped $Cs_2NaBiCl_6$ NCs synthesized at 200 °C.

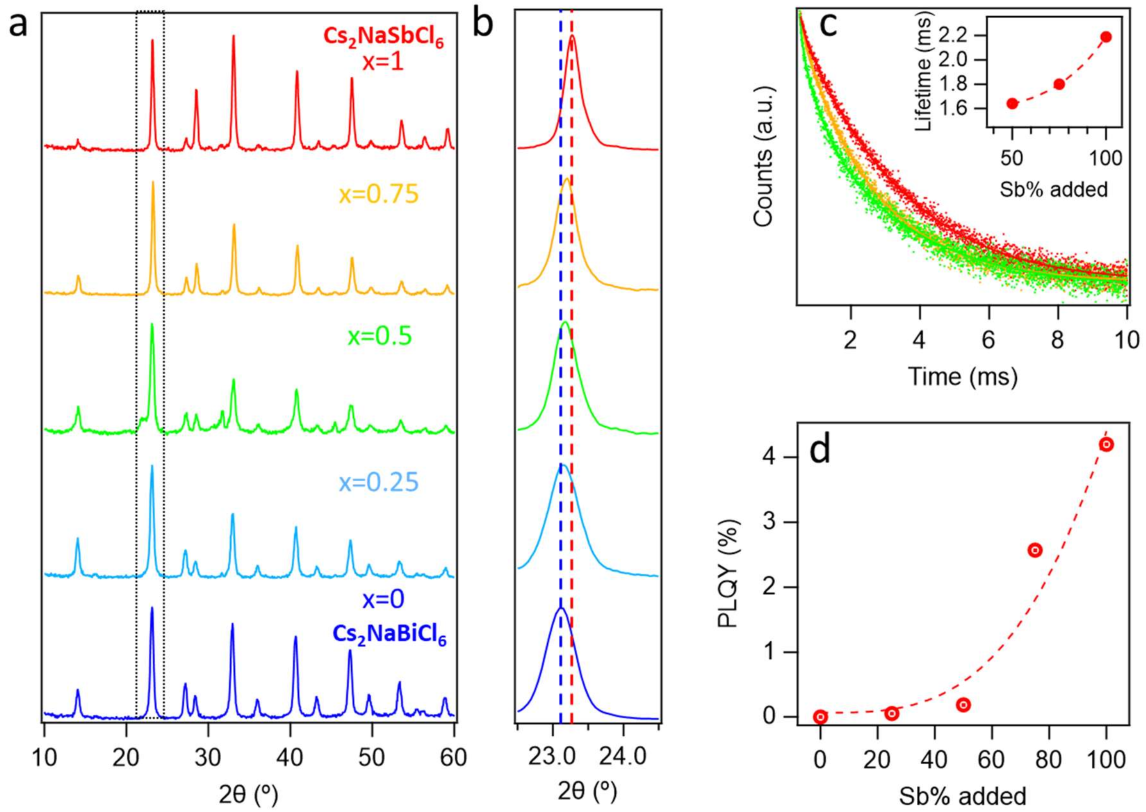

Figure S8. (a) XRD pattern of Nd^{3+} doped $Cs_2NaSb_xBi_{1-x}Cl_6$ NCs. (b) Zoomed-in XRD patterns showing shifting of the (220) diffraction peak. (c) TR-PL spectra and inset showing average PL lifetime of Nd^{3+} doped $Cs_2NaSb_xBi_{1-x}Cl_6$ (x = 0.5, 0.75 and 1) NCs for the NIR emission at 1065 nm. (d) NIR PL QYs of Nd^{3+} doped $Cs_2NaSb_xBi_{1-x}Cl_6$ NCs.

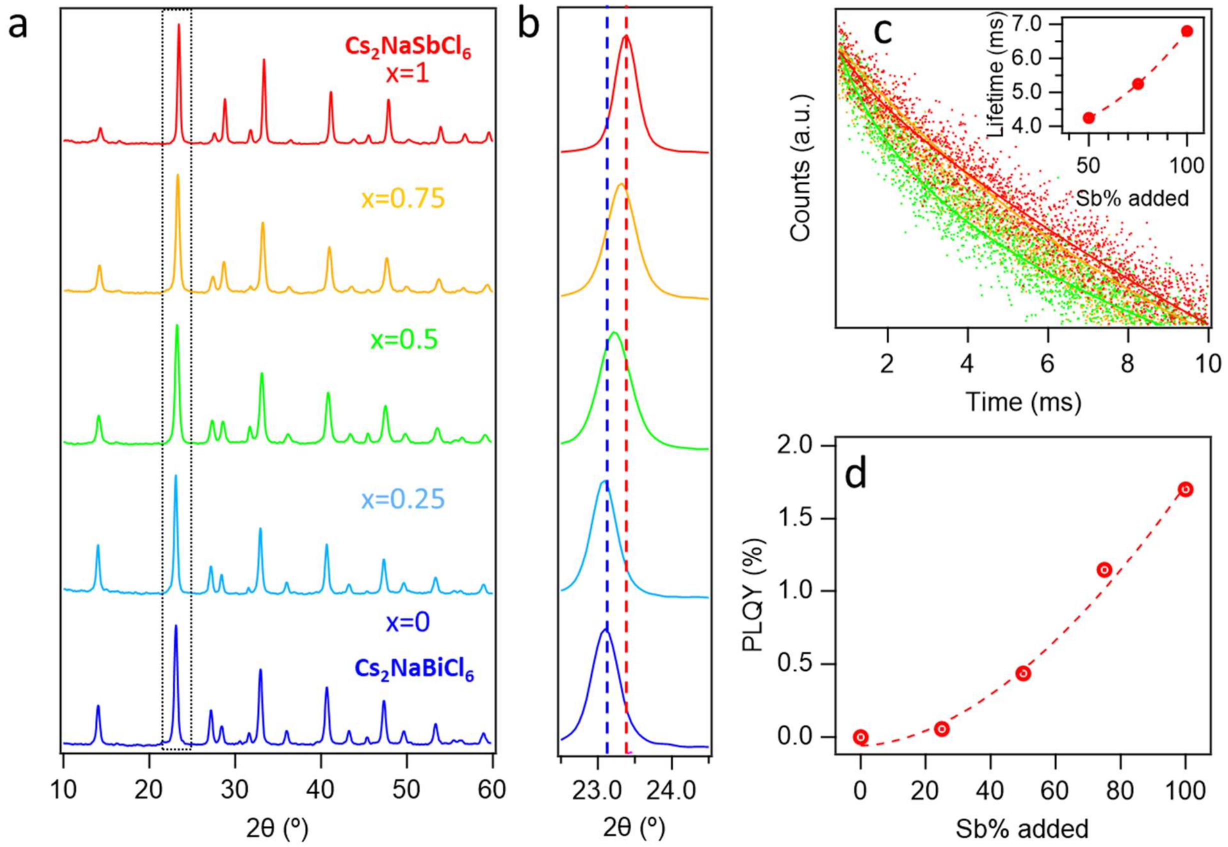

Figure S9. (a) XRD pattern of Er^{3+} doped $Cs_2NaSb_xBi_{1-x}Cl_6$ NCs. (b) Zoomed-in XRD patterns showing shifting of the (220) diffraction peak. (c) TR-PL spectra and inset showing average PL lifetime of Er^{3+} doped $Cs_2NaSb_xBi_{1-x}Cl_6$ (x=0.5, 0.75 and 1) NCs for the NIR emission at 1650 nm. (d) NIR PL QYs of Er^{3+} doped $Cs_2NaSb_xBi_{1-x}Cl_6$ NCs.

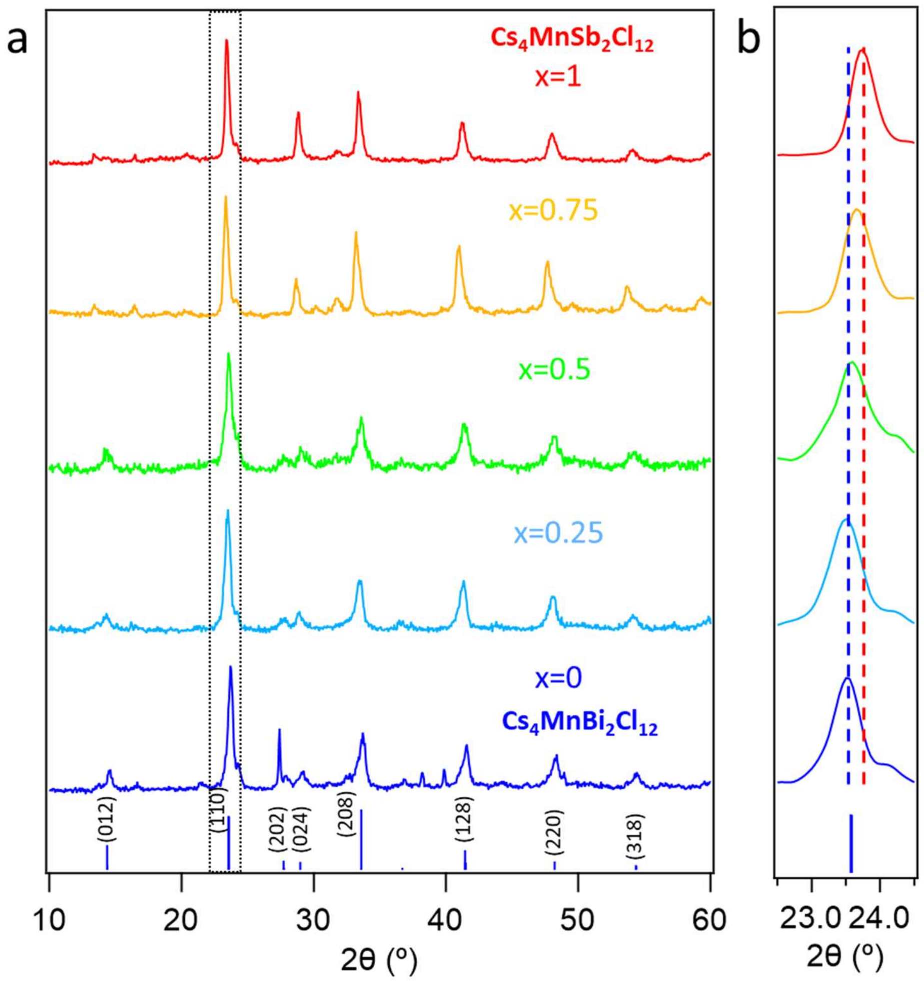

Figure S10. (a) XRD pattern of Yb^{3+} doped $Cs_4MnSb_{2x}Bi_{2-2x}Cl_{12}$ NCs. (b) Zoomed-in XRD patterns showing shifting of the (110) diffraction peak.

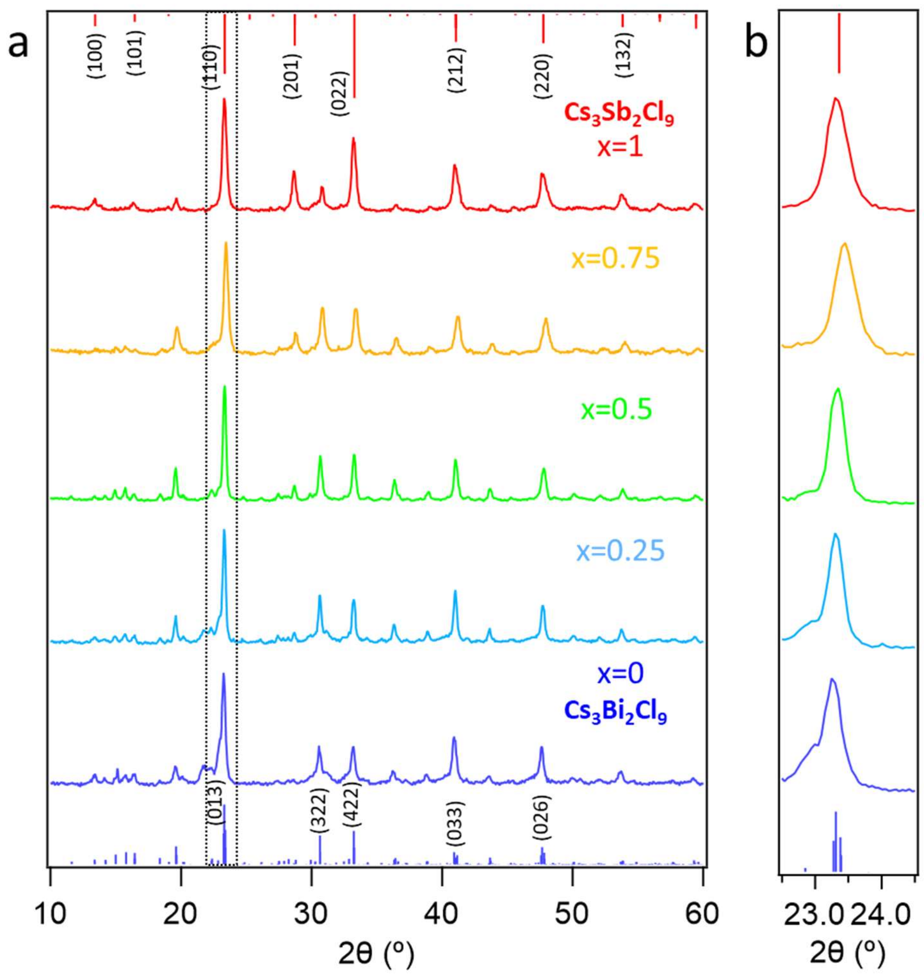

Figure S11. (a) XRD pattern of Yb^{3+} doped $Cs_3Sb_{2x}Bi_{2-2x}Cl_9$ NCs. (b) Zoomed-in XRD patterns showing shifting of the (013) diffraction peak of Yb^{3+} doped Cs2NaBiCl6 by alloying Sb^{3+}.

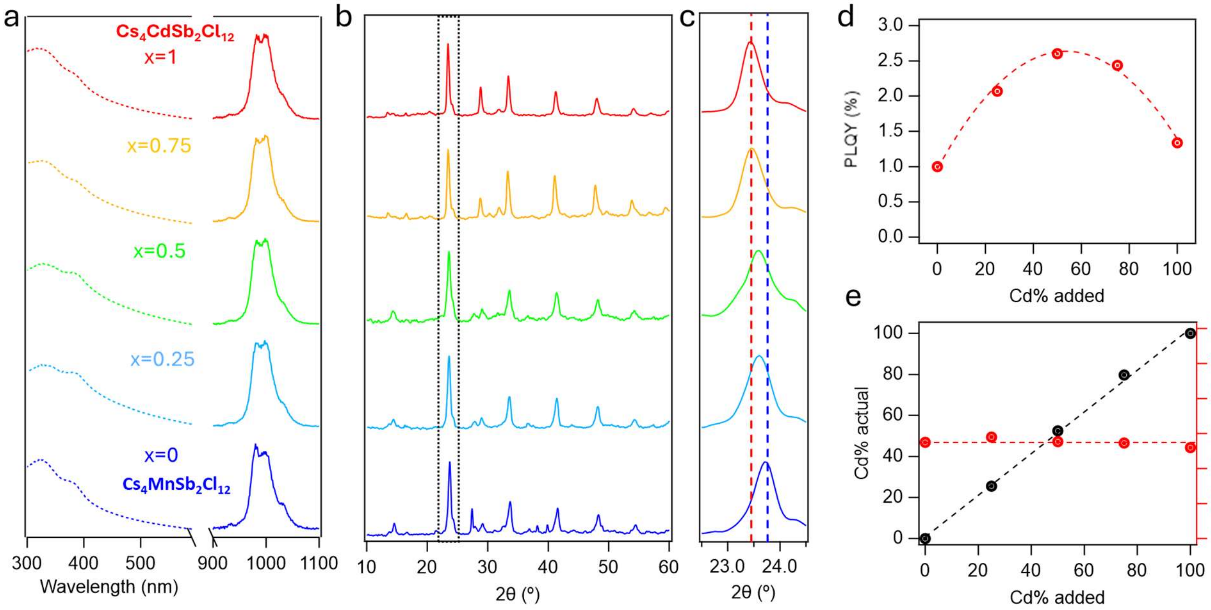

Figure S12. (a) Normalized absorption and NIR PL emissions, (b) XRD, (c) zoomed-in XRD patterns showing shifting of the (110) diffraction peak, (d) NIR PL QYs, and (e) actual Sb^{3+} and Yb^{3+} content of Yb^{3+} doped $Cs_4Mn_{1-x}Cd_xSb_2Cl_{12}$ NCs.

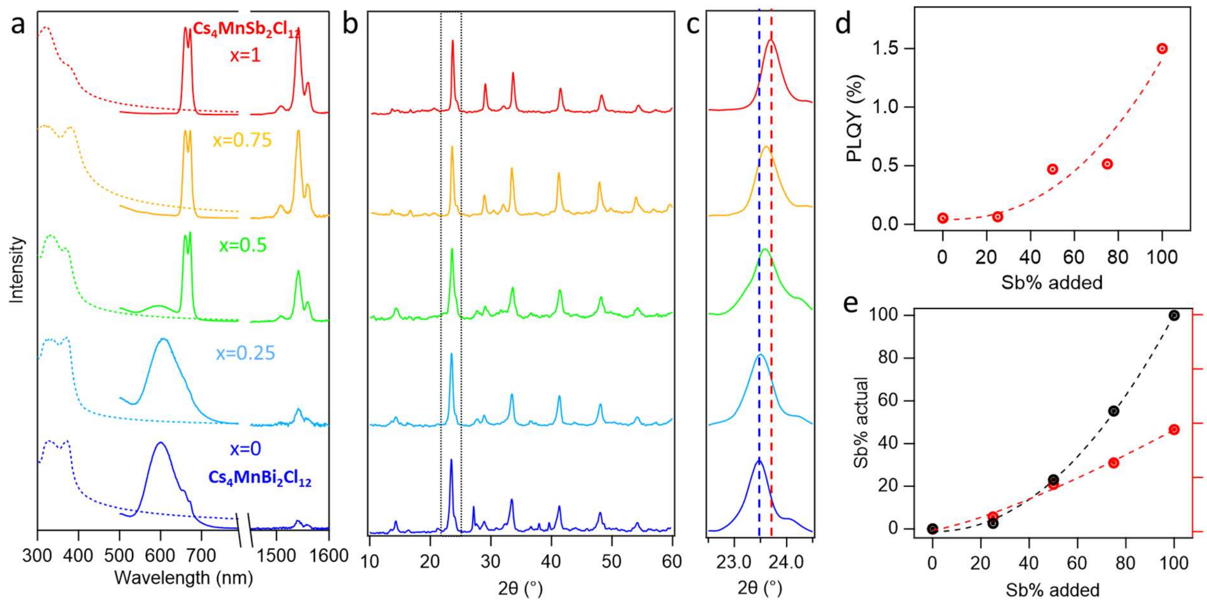

Figure S13. (a) Normalized absorption and NIR PL emissions, (b) XRD, (c) zoomed-in XRD patterns showing shifting of the (110) diffraction peak, (d) NIR PL QYs, and (e) actual Sb^{3+} and Er^{3+} content of Er^{3+} doped $Cs_4Mn_{1-x}Cd_xSb_2Cl_{12}$ NCs by ICP measurements.

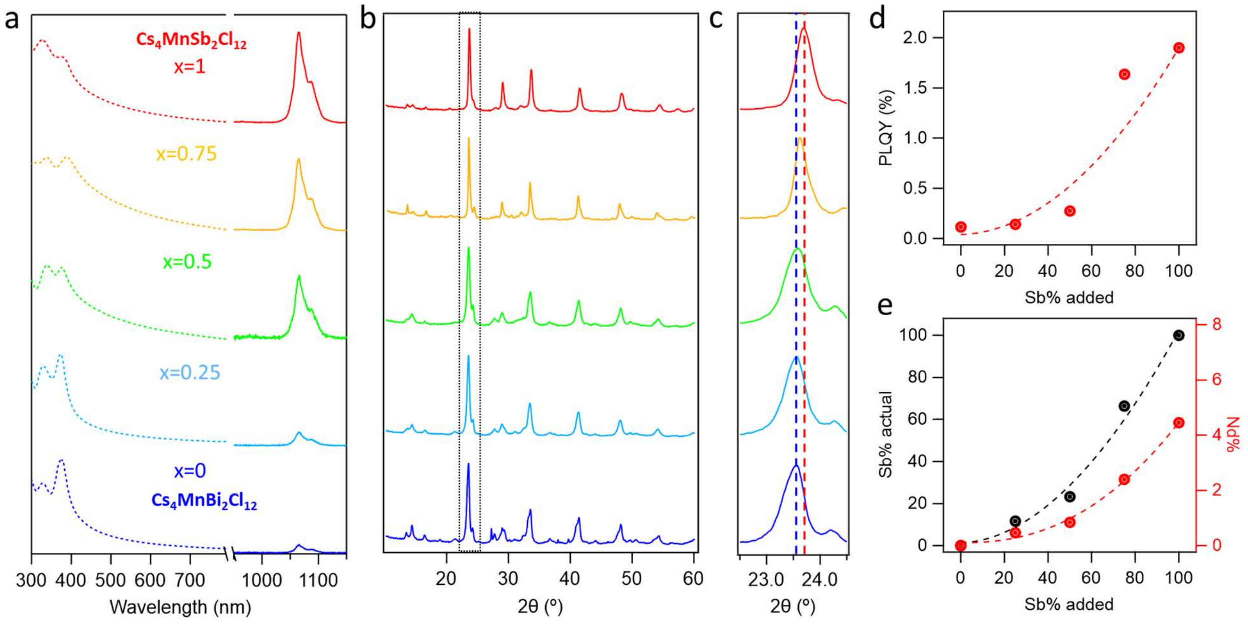

Figure S14. (a) Normalized absorption and NIR PL emissions, (b) XRD, (c) zoomed-in XRD patterns showing shifting of the (110) diffraction peak, (d) NIR PL QYs, and (e) actual Sb^{3+} and Nd^{3+} content of Nd^{3+} doped $Cs_4Mn_{1-x}Cd_xSb_2Cl_{12}$ NCs by ICP measurements.

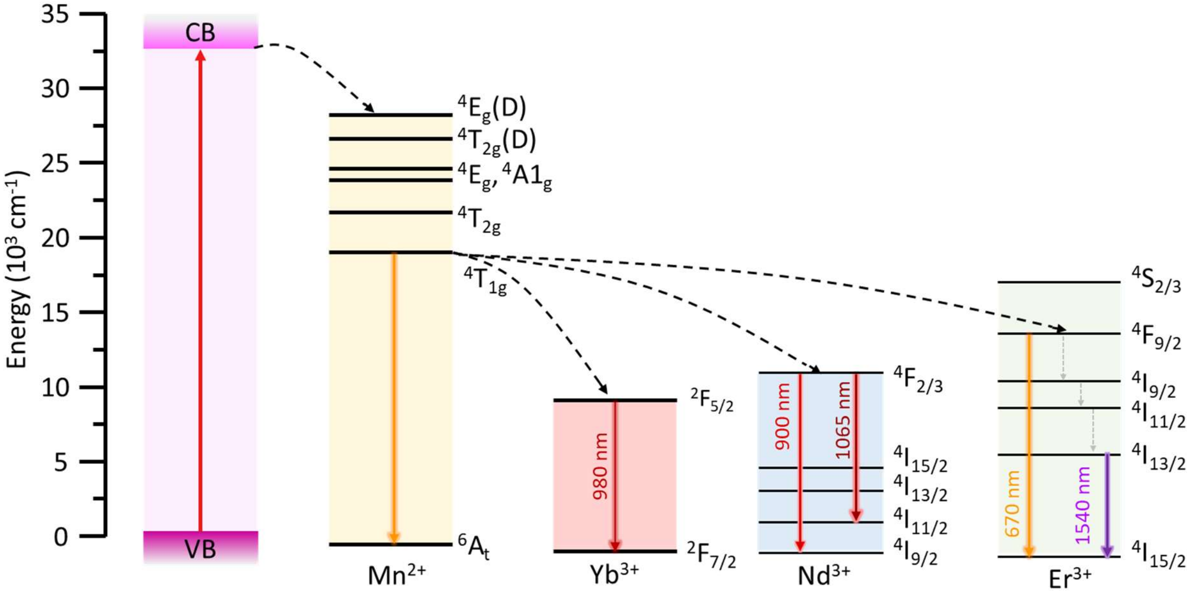

Figure S15. (a) Schematic illustration of energy level alignment and energy transfer pathway for Ln^{3+} doped $Cs_4MnSb_2Cl_{12}$ NCs.

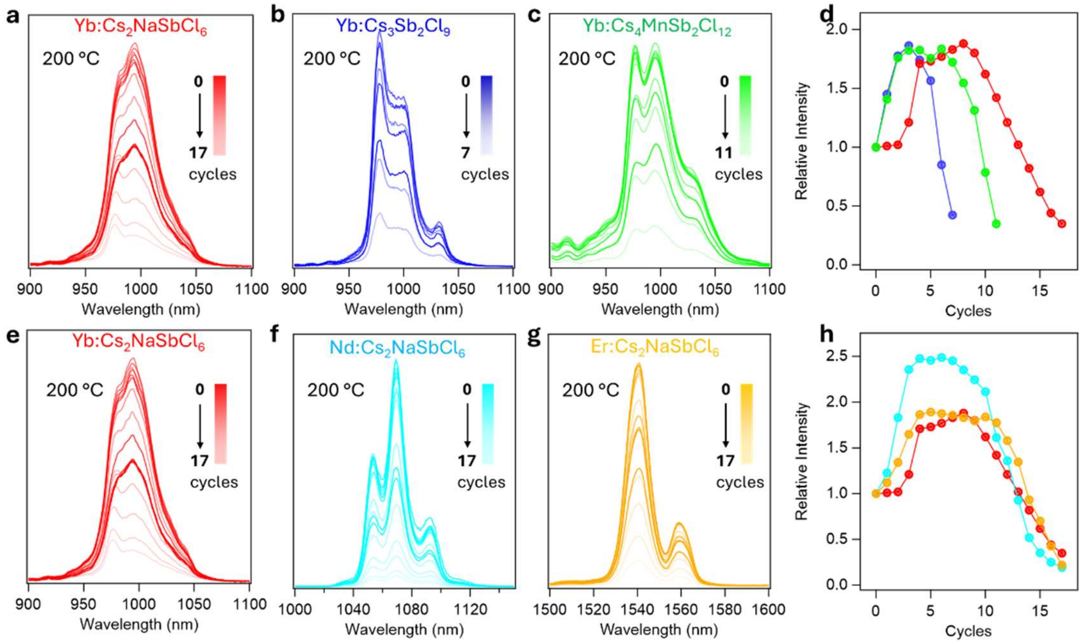

Figure S16. Thermal stability at 200 °C of Yb^{3+} doped (a) $Cs_2NaSbCl_6$, (b) $Cs_3Sb_2Cl_9$, and (c) $Cs_4MnSb_2Cl_{12}$ NCs, and (d) their relative intensities as a function of heating cycles (10 min per cycle). Thermal stability at 200 °C of Yb^{3+}, Nd^{3+} and Er^{3+}-doped $Cs_2NaSbCl_6$ (e-g), and (h) their relative intensities as a function of heating cycles (10 min per cycle).

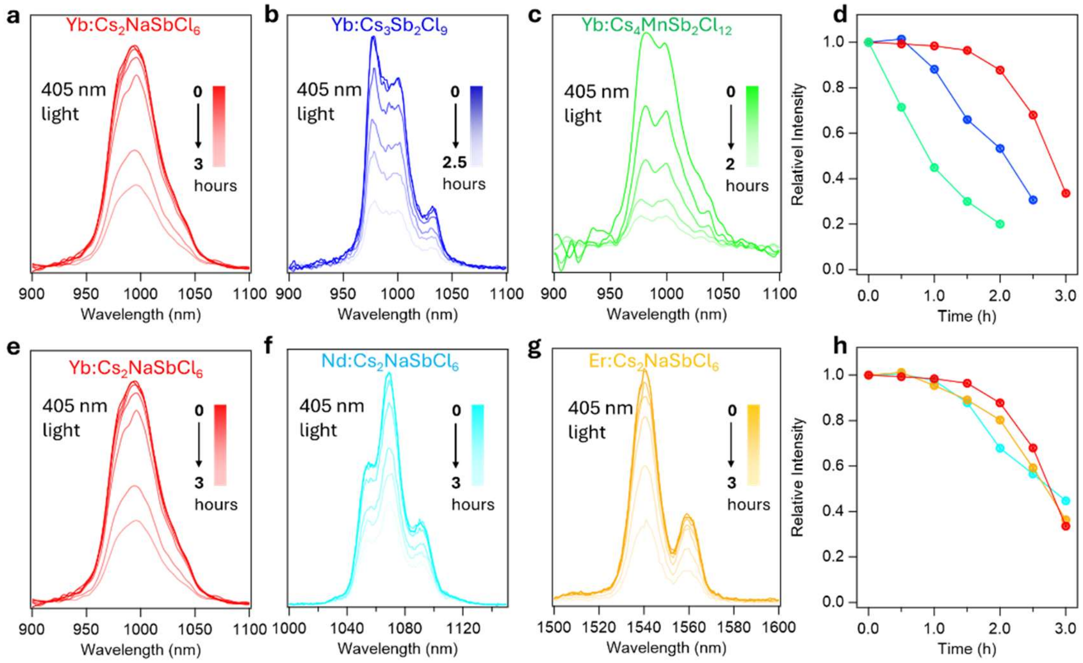

Figure S17. Blue light stability of Yb^{3+} doped (a) $Cs_2NaSbCl_6$, (b) $Cs_3Sb_2Cl_9$, and (c) $Cs_4MnSb_2Cl_{12}$ NCs, and (d) their relative intensities as a function of light irradiation time. UV-light stability of Yb^{3+}, Nd^{3+} and Er^{3+} doped $Cs_2NaSbCl_6$ (e-g), and (h) their relative intensities as a function of light irradiation time.

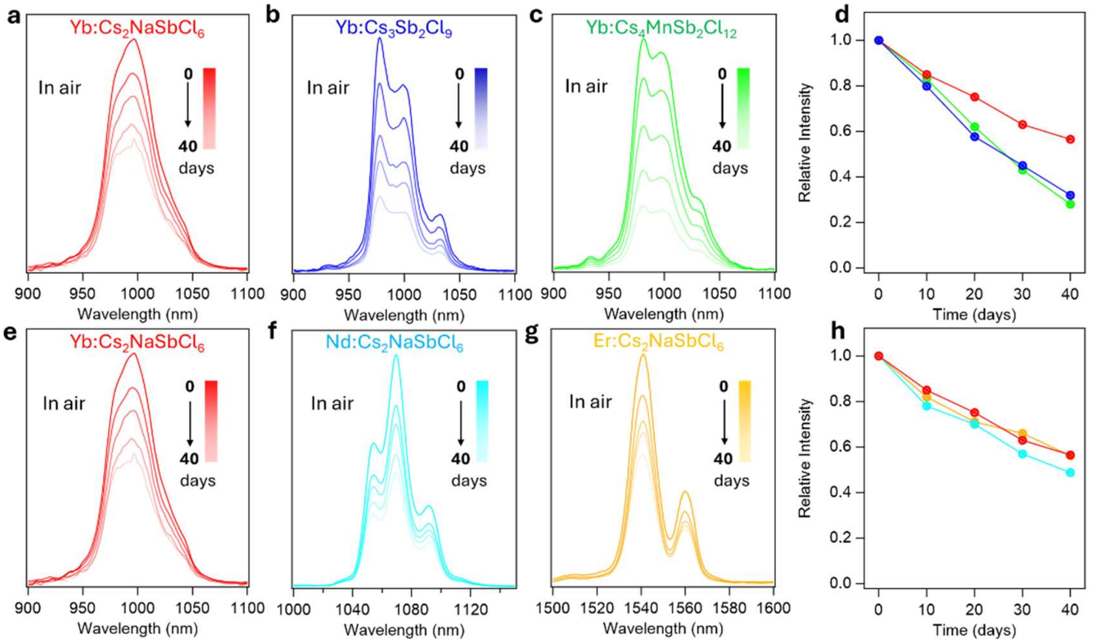

Figure S18. Ambient/air stability (T = 20 °C, RH = 70%) of Yb^{3+} doped (a) $Cs_2NaSbCl_6$, (b) $Cs_3Sb_2Cl_9$ and (c) $Cs_4MnSb_2Cl_{12}$ NCs, and (d) their relative intensities vs light irradiation time. Ambient stability of Yb^{3+}, Nd^{3+} and Er^{3+}-doped $Cs_2NaSbCl_6$, (e-g) and (h) their relative intensities as a function of time.

Table S2. DFT energy calculations for Ln^{3+} ions doped in $[SbCl_6]^{3-}$ and $[BiCl_6]^{3-}$ O_h sites with the bond length of 2.683 and 2.647 Å, respectively, using the B3LYP functional and def2-TZVP basis set with ECP for the lanthanide ions.

	**Bi site (eV)**	**Sb site (eV)**	**Relative Stability of Sb and Bi sites (eV)**	
**$[NdCl_6]^{3-}$**	-90424.80545	-90426.3545	1.549	**Sb favored**
**$[ErCl_6]^{3-}$**	-102780.0774	-102780.1823	0.105	**Sb favored**
**$[YbCl_6]^{3-}$**	-106697.6553	-106697.7456	0.090	**Sb favored**

Reference.

1. Lee, W.; Choi, D.; Kim, S., Colloidal Synthesis of Shape-Controlled Cs_2NaBiX_6 (X = Cl, Br) Double Perovskite Nanocrystals: Discrete Optical Transition by Non-Bonding Characters and Energy Transfer to Mn Dopants. *Chem. Mater.* **2020,** *32* (16), 6864-6874.

2. Cai, T.; Shi, W.; Wu, R.; Chu, C.; Jin, N.; Wang, J.; Zheng, W.; Wang, X.; Chen, O., Lanthanide Doping into All-Inorganic Heterometallic Halide Layered Double Perovskite Nanocrystals for Multimodal Visible and Near-Infrared Emission. *J. Am. Chem. Soc.* **2024,** *146* (5), 3200-3209.
